# Cytosolic Access of *Mycobacterium tuberculosis*: Critical Impact of Phagosomal Acidification Control and Demonstration of Occurrence *In Vivo*

**DOI:** 10.1371/journal.ppat.1004650

**Published:** 2015-02-06

**Authors:** Roxane Simeone, Fadel Sayes, Okryul Song, Matthias I. Gröschel, Priscille Brodin, Roland Brosch, Laleh Majlessi

**Affiliations:** 1 Institut Pasteur, Unit for Integrated Mycobacterial Pathogenomics, Institut Pasteur, Paris, France; 2 Inserm U1019, CNRS UMR8204, Université de Lille–Nord de France, Institut Pasteur de Lille, Center for Infection and Immunity, Lille, France; New Jersey Medical School, UNITED STATES

## Abstract

*Mycobacterium tuberculosis* (*Mtb*) uses efficient strategies to evade the eradication by professional phagocytes, involving—as recently confirmed—escape from phagosomal confinement. While *Mtb* determinants, such as the ESX-1 type VII secretion system, that contribute to this phenomenon are known, the host cell factors governing this important biological process are yet unexplored. Using a newly developed flow-cytometric approach for *Mtb*, we show that macrophages expressing the phagosomal bivalent cation transporter Nramp-1, are much less susceptible to phagosomal rupture. Together with results from the use of the phagosome acidification inhibitor bafilomycin, we demonstrate that restriction of phagosomal acidification is a prerequisite for mycobacterial phagosomal rupture and cytosolic contact. Using different *in vivo* approaches including an enrichment and screen for tracking rare infected phagocytes carrying the CD45.1 hematopoietic allelic marker, we here provide first and unique evidence of *M. tuberculosis*-mediated phagosomal rupture in mouse spleen and lungs and in numerous phagocyte types. Our results, linking the ability of restriction of phagosome acidification to cytosolic access, provide an important conceptual advance for our knowledge on host processes targeted by *Mtb* evasion strategies.

## Author Summary

The intracellular fate of the agent of the human tuberculosis agent in phagocytes is a question of great biological relevance. Among the mycobacterial survival strategies, the escape of *Mycobacterium tuberculosis* from phagosomes has been subject of scientific debate for a long time. However, technically improved methods recently reinforced the occurrence of this phenomenon. Here, we focused on the host factors involved in phagosomal rupture and provide first and singular evidence of *M*. *tuberculosis*-mediated phagosomal rupture *in vivo* in mouse lungs and inside the granuloma. We show that partial blockage of phagosomal acidification, induced by mycobacteria, is a prerequisite for efficient vacuolar breakage by *M*. *tuberculosis* and link maturation arrest, cytosolic contact and the corresponding immune responses. From our results we conclude that vacuolar breakage induced by *M*. *tuberculosis* is not an *ex vivo* artifact of cell cultures, but an important process that occurs inside infected phagocytes within organs during several days that strongly determines the outcome of infection with this key pathogen.

## Introduction

The pathogenic potential of *Mycobacterium tuberculosis* (*Mtb*), the etiologic agent of human tuberculosis (TB), depends largely on the type VII secretion system ESX-1 [1,2], which is responsible for the secretion of the 6-kDa Early Secreted Antigenic Target (ESAT-6), its protein partner, the 10-kDa Culture Filtrate Protein (CFP-10), and several ESX-1 associated proteins (Esps) [3,4]. ESX-1 secretion is evolutionary conserved in most members of the *M*. *tuberculosis* complex [5], and the more distantly related tubercle bacilli of the *Mycobacterium canettii* clade [6,7], as well as in some non-tuberculous mycobacteria such as *Mycobacterium marinum* [8]. This secretion system governs numerous aspects of interaction between pathogenic mycobacteria and the host cell [1,2], including membrane-damaging activity [9–11], thought to be implicated in phagosomal escape at later stages of infection [12–16]. Although this phenomenon is a matter of debate [2,17–20], by use of a single-cell Fluorescence Resonance Energy Transfer (FRET)-based technology [21], we recently demonstrated that ESX-1-proficient *Mtb* and recombinant *Mycobacterium bovis* BCG::ESX-1 were able to induce phagosome rupture in human THP-1 macrophage (MΦ)-like cells [15]. This assay uses the ability of the surface-exposed BlaC β-lactamase of *Mtb* [22,23] to cleave the FRET substrate CCF-4, which consists of a cephalosporin core linking 7-hydroxycoumarin to fluorescein that has also been used for exploring effector injection and intracellular localization of Gram-negative bacteria [21,24,25]. The ESX-1-induced rupture of the phagosomal membrane, which results in the exit of mycobacterial products from the endosomal pathway and in extra-phagosomal localization of bacilli [13–16] is of relevance for the outcome of the immune control and bacterial dissemination [26–29]. Phagosomes are reported to be specialized platforms for pathogen recognition [30] and there is also growing evidence of a link between the functionality of the ESX-1 secretion system and the presence of mycobacteria-associated molecular patterns in the host cytosol. Peptidoglycans [31,32] and extracellular mycobacterial DNA [33] were reported to be sensed by the cytosolic receptors of the innate system with multiple biological consequences. Indeed, the *Mtb*-mediated induction of Nucleotide binding Oligomerization Domain (NOD)-Like Receptor pathways, i.e., NOD2 / Receptor-interacting protein 2 kinase (Rip2) / TANK-Binding Kinase 1 (TBK1) / Interferon regulatory factor (Irf) 5, is responsible for a significant part of type I interferon (IFN) production [31,32]. On the other hand, the signaling through the Stimulator of IFN Genes (STING) / TBK1 / Irf3 pathway [33] leads to a type I IFN signature on which depends the expression of CCL5, CXCL10 and Nitric Oxide Synthase 2 [34,35]. The formation of Nucleotide-binding domain and Leucin-rich Repeat pyrin–containing Protein-3 (NLRP-3) / ASC (Apoptosis-associated Speck-like protein containing a carboxy-terminal CARD) / caspase-1 inflammasome complex, is required in humans for the processing of the pro-IL-1β into biologically active pleïotropic immune mediator IL-1β following *Mtb* infection [36,37]. Moreover, the ubiquitination of *Mtb* prior to its delivery to the autophagic machinery also necessitates the ESX-1-dependent translocation of extracellular *Mtb* DNA to the cytosol [16,33,38,39]. Thus, the events arising from mycobacterial cytoplasmic access may substantially influence both the immune control of *Mtb* and the inflammation-induced tissue damage.

The impact of selected components of the ESX-1 system on phagosomal rupture has recently been assessed [13,15,16], however, other potential intervening factors, including those from the host cell remain largely unexplored. Here, we have investigated the host parameters modulating the *Mtb*-mediated vacuolar breakage, by developing a CCF-4 FRET-based approach that can be used for the study of *Mtb*-infected cells by flow cytometry. This approach, which permits to combine the detection of phagosomal rupture with the analysis of numerous host cell phenotypic and functional parameters, allowed us to explore multiple phagocyte types, including those isolated from mouse airways. Our results provide first and unique evidence that *Mtb*-induced phagosomal rupture does occur *in vivo* inside the lungs and spleens of infected experimental animals and lasts over several days. Moreover, we here explore the impact of vacuolar acidification that constitutes a fundamental cellular defense mechanism [40] and demonstrate that the characteristic partial prevention of phagosomal acidification by *Mtb* is a prerequisite for phagosomal escape of the pathogen. Our study thus reveals novel details and presents a refined model of cellular events during infection with *Mtb*.

## Results

### ESX-1-dependent *Mtb*-mediated phagosomal rupture detected by FRET-based flow cytometry

**Fig 1:**
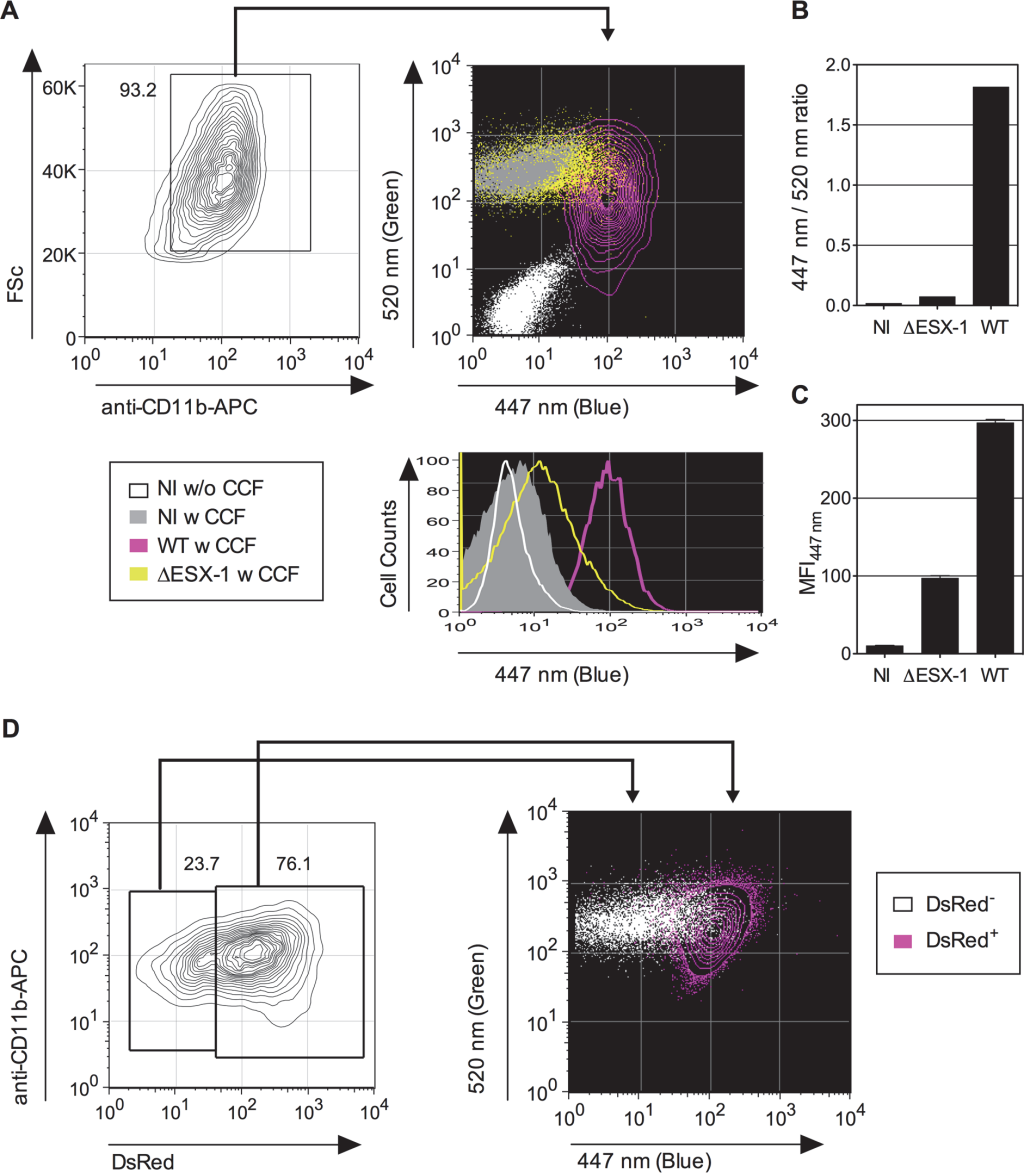
Detection of *Mtb*-mediated phagosome disruption by flow cytometry. (A) Phagosomal rupture detected by CCF-4 FRET-based flow cytometry. Differentiated THP-1 cells were infected with *Mtb*, WT or ΔESX-1 strain (MOI = 1); NI = not infected. At 4 dpi, cells were successively stained with CCF-4 and anti-CD11b mAb, fixed, and their green (520 nm) *vs*. blue (447 nm) fluorescent signals were analyzed after gating on CD11b^+^ cells. Results are depicted as signal overlays of different groups as dot or contour blots. (B-C) Shown are ratios of MFI_447 nm_ / MFI_520 nm_ (B) and blue MFI_447 nm_ (C), calculated as described in Materials and Methods. (D) Differentiated THP-1 cells were infected with DsRed-expressing *Mtb* H37Rv strain (MOI = 0.3). At 4 dpi, cells were stained as in A. The cells having phagocytosed DsRed-*Mtb* (DsRed^+^) were first gated for their red signal and their green *vs*. blue CCF-4 signals were compared to the cells in the same culture that had not engulfed DsRed-*Mtb* (DsRed^-^). The results are representative of at least 3 independent experiments.

To evaluate mycobacteria-mediated phagosomal rupture in different phagocyte types and different physiological contexts, we adapted the previously used microscopy-based CCF-4 FRET technique [15] for flow cytometry. The latter approach not only allows monitoring of bacteria-induced phagosomal rupture or tracking of endosome-to-cytosol antigen translocation [25,41], but also permits the simultaneous inspection of surface markers and analysis of hundreds of thousands of host cells. At first, we infected differentiated THP-1 cells at a multiplicity of infection (MOI) of 1 either with *Mtb* H37Rv WT or the isogenic ΔESX-1 derivative, *Mtb* H37Rv-ΔRD1 [10], which both display similar β-lactamase activity [15]. These THP-1 cells were then incubated with CCF-4-AM, an esterified, lipophilic form of the CCF-4 substrate that can readily enter into cells, where it is converted by endogenous cytoplasmic esterases into negatively charged CCF-4, which is retained in the cytosol and emits green fluorescence (500–550 nm) upon stimulation at 320–380 nm, due to FRET from the coumarin moiety to the fluoroscein part (S1 Fig.). In the case of *Mtb*-induced phagosomal rupture, cleavage of CCF-4 by the intrinsic *Mtb* BlaC β-lactamase leads to loss of FRET and a change of the CCF-4 emission spectrum from green to blue coumarin fluorescence (410–470 nm). As depicted in Fig. 1A, the CCF-4 emission signals of CD11b^+^ gated THP-1 cells, infected with wild-type (WT) *Mtb* H37Rv, showed a marked shift of the CCF-4 emission towards blue at 4 days post infection (dpi). In contrast, a much weaker shift of the CCF-4 spectrum was observed for *Mtb* ΔESX-1-infected cells, validating our experimental setup and confirming the fundamental virulence differences between the used ESX-1-proficient and ESX-1–deficient *Mtb* strains [10,15]. The residual blue shift in *Mtb* ΔESX-1-infected cells relative to non-infected cells is likely a consequence of paraformaldehyde (PFA) fixation prior to signal acquisition (S2A–B Fig.). These results were further corroborated by ratios of Mean Fluorescence Intensities (MFI) of blue *vs*. green signals (Fig. 1B), and blue MFI_447 nm_ (Fig. 1C). Moreover, we also used fluorescent *Mtb* (DsRed-*Mtb* H37Rv) to infect THP-1 cells, at a weaker initial dose (MOI = 0.3), and thereby observed that the CCF-4 blue emission shift selectively occurred in cells that had engulfed the bacteria (Fig. 1D). This approach thus allowed a quantitative study of phagosomal rupture in host cells that have engulfed *Mtb*, and whose subtype can be identified/determined by staining of the specific surface markers. Hence, our experimental setup was adapted to be used for various cell types and physiological situations, including the detection of vacuolar rupture in rare (infected) cells that were dispersed in a large and heterogeneous cell background population.

### *Mtb*-induced phagosomal rupture in dendritic cells and macrophages, relationship with the infection rate and cell necrosis

**Fig 2:**
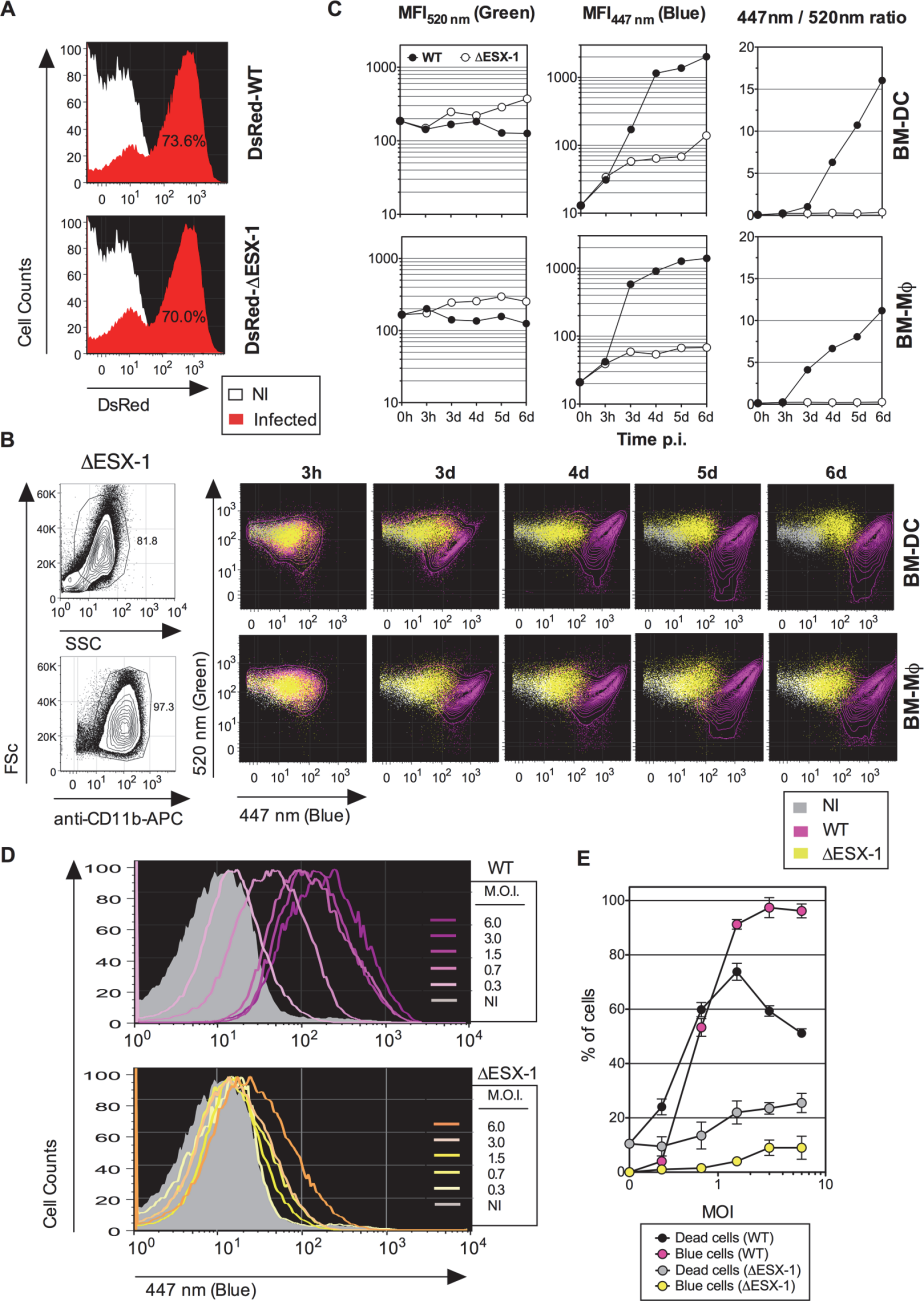
*Mtb*-mediated phagosome disruption in different phagocyte types, relationship with infection dose and cell death. (A) Comparative infectivity of WT and ΔESX-1 *Mtb*. BM-DC were infected with DsRed-WT or-ΔESX-1 strain and the red fluorescence was assessed by cytometry at 1 dpi. Percentages of cells having phagocytized DsRed-mycobacteria are indicated. (B) Detection of phagosomal rupture subsequent to infection with WT or ΔESX-1 strains, as determined by green *vs*. blue CCF-4 signals in BM-DC or BM-MΦ, infected with untagged *Mtb*, WT or ΔESX-1 (MOI = 1) at different time points, as detected after exclusion of cell debris and free bacteria by FSc/SSc gating and inclusion of CD11b^+^ cells. (C) MFI_520 nm_, MFI_447 nm_ and MFI_447 nm_/MFI_520 nm_ ratios in infected BM-DC or BM-MΦ at different time points. (D) Phagosomal rupture, monitored at 4 dpi by CCF-4 staining, in BM-DC infected with different MOI of WT or ΔESX-1 *Mtb*. (E) Percentages of dead cells, as determined by the use of Pacific Blue Dead/Live reagents, compared to those of cells displaying a CCF-4 blue shift. Due to the emission overlap of CCF-4-Coumarin and Pacific Blue fluorochromes, the two different assays were performed in separate tubes in parallel, in cells from the same BM-DC cultures. The results are representative of 2 independent experiments. Of note, the decrease of the dead cell percentage for WT *Mtb* at very high MOI is likely due to generation of cell debris, not anymore measurable by cytometry.

Dendritic cells (DC) and MΦ do not play the same roles during the infection. DCs that have engulfed *Mtb*, are more prone to process and present pathogen-derived antigens and to prime T cells than *Mtb*-laden MΦ which are thought to initiate the inflammatory program and are considered as long-term *Mtb* reservoirs. We thus comparatively evaluated the potential of *Mtb* to induce phagosome rupture in bone-marrow-derived (BM)-DC and -MΦ At first, by using fluorescent DsRed WT and ΔESX-1 *Mtb* variants, we showed similar uptake and infectivity of both strains at the beginning of the infection (Fig. 2A). Infection of BM-DC and BM-MΦ with WT *Mtb* then resulted in a strong blue shift at 3 dpi and thereafter, whereas for cells infected with the ΔESX-1 *Mtb* strain only a minor blue shift was detected (Fig. 2B). The relatively stable CCF-4 green signal and its progressively increasing blue shift for WT *Mtb* resulted in a blue/green ratio of 15 in BM-DC and 10 in BM-MΦ respectively, at 6 dpi (Fig. 2C). Similar as observed for THP-1 cells (Fig. 1D), infection of BM-DC with DsRed expressing *Mtb* showed that cells, which had engulfed DsRed *Mtb*, progressively increased their CCF4 blue shift over the observation period of 3 to 5 dpi (S3 Fig.). Together, these results suggest that ESX-1-dependent, *Mtb*-induced phagosomal rupture does occur in DC and MΦ.

To ascertain that the absence of FRET inhibition in cells infected with the ΔESX-1 *Mtb* mutant was not due to other molecular reasons than the absence of the ESX-1 secretion system, we complemented the *Mtb* ΔESX-1 strain with the integrative cosmid p2F9, containing 32 kb of the ESX-1 encoding genomic region from *Mtb* H37Rv [42]. This complementation reconstituted the ability of the resulting strain to induce phagosomal rupture, and thereby validated the ΔESX-1 mutants used throughout this study (S2C Fig.).

When uncontrolled inside the host cell, *Mtb* infection may lead to necrosis [27,43], which could theoretically allow exchanges between phagosome and cytosol and thereby establish a contact between mycobacterial β-lactamase located within the phagosome and CCF-4 located inside the cytosol. To investigate this key question, we determined whether the cytosolic access of *Mtb* was a consequence of host cell necrosis. In a dose-response experiment, changes in the FRET signal for the *Mtb* WT strain were seen as a function of the MOI (Fig. 2D). Except for an MOI below 1, the proportions of BM-DC displaying FRET inhibition were higher than the percentages of necrotic cells (Fig. 2E). In contrast, BM-DC infected with *Mtb* ΔESX-1 at the same MOIs displayed much weaker CCF-4 blue shifts. These data suggest that ESX-1-mediated phagosomal rupture progressively occurs in phagocytes in an MOI-dependent manner and that the resultant presence of mycobacterial β-lactamase activity in the host cell cytosol does not arise from host cell necrosis but rather precedes cell death.

### Early minor levels of phagosome disruption and their full proportionality with type I IFN production

**Fig 3:**
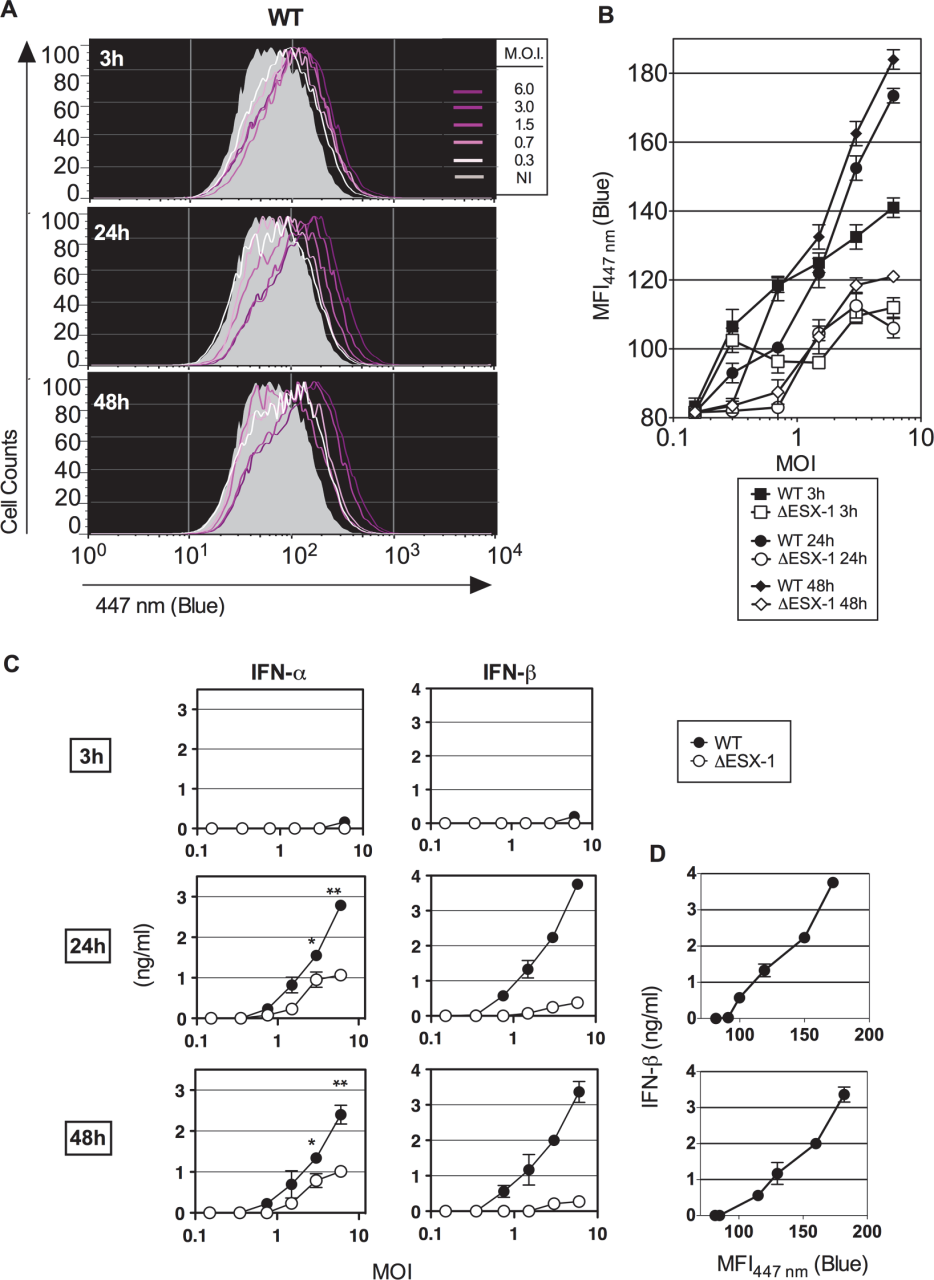
Early *Mtb*-mediated phagosomal rupture, relationship with secretion of type I IFNs. (A) BM-DC were infected with *Mtb* WT at different MOI and the phagosomal rupture was assessed by CCF-4 staining at early time points of 3, 24 and 48 hpi. (B) MFI_447 nm_ of the infected cells at each time point and for different MOI of WT or ΔESX-1 strain. (C) IFN-α and-β concentrations, as quantified in the supernatants of the same infected cells by ELISA. *, ** = statistically significant, *p*<0.01 or *p*<0.001, respectively, as determined by the Student's *t* test. (D) Linear relationship between the amounts of IFN-β produced and *Mtb*-induced phagosomal rupture in BM-DC. Shown are representative data from 2 independent experiments.

So far, *Mtb*-induced phagosomal rupture has only been observed at later stages of infection, *i*.*e*, 3–5 dpi, a kinetic situation, which cannot explain the very early, ESX-1-dependent release of type I IFNs or IL-1β, that requires recognition of mycobacterial components by the host cytosolic sensors [44]. However, our highly sensitive approach allowed now detection of minor levels of FRET inhibition indicated by enhanced MFI_447 nm_ (blue), as early as 3 hours post infection (hpi) with WT *Mtb* (Fig. 3A-B). The blue shift then progressively increased at 24 and 48 hpi, although it remained still low compared to values obtained for later time points (Fig. 2B-C). Comparison of these results with those from infection experiments using the *Mtb* ΔESX-1 deletion mutant, which overall showed much lower MFI_447nm_ (blue) values (Fig. 3B), suggests that *Mtb*-mediated phagosomal rupture begins already at such early time-points, likely caused by initial ESX-1-induced pore forming activity, and progresses into stronger phagosomal disassembly over time. These findings suggest that the time during which the *Mtb*-infected host cell displays phagosomal rupture and *Mtb* cytosolic access, prior to host cell death, is longer than previously estimated [15].

Considering the long *Mtb* replication time of ≈ 20h, such early initiation of *Mtb*-mediated phagosomal rupture suggests that this phenomenon does not depend on bacterial replication, but on the functions of the implicated bacterial virulence factors. The levels of phagosome disruption were entirely proportional to the amounts of secreted IFN-β (Fig. 3C). A partially ESX-1-dependent increase in the IFN-α secretion was also detected, which might be linked to the induction of Irf7 subsequent to IFN-β induction [45]. Therefore, minute levels of early phagosomal rupture are in direct correlation with the kinetics of the induction of type I IFN production. In contrast, no differences were found between ESX-1-proficient and ΔESX-1 *Mtb* strains when IL-1β secretion was studied (S4 Fig.), which is consistent with the inflammasome/caspase-1-independent IL-1β secretion in mice during *Mtb* infection [46] and which is different to the situation in humans [36].

### Link between the phagosomal environment and the ability to induce phagosomal rupture

**Fig 4:**
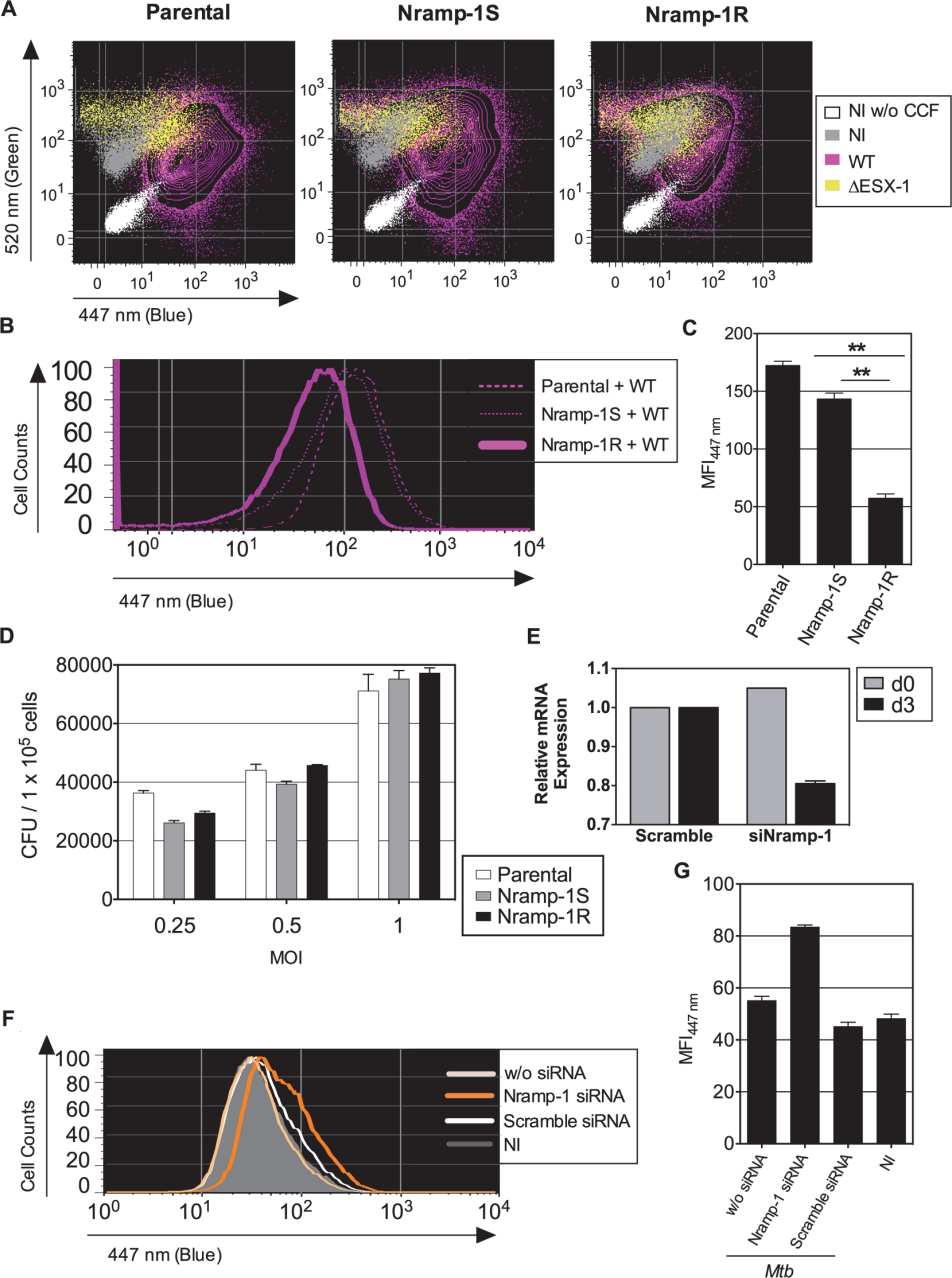
Host Nramp-1 transporter counteracts the phagosomal rupture in *Mtb*-infected MΦ. (A) Raw264.7 cells, parental or transfected with non-functional *nramp-1S* or functional *nramp-1R* allele, were infected with *Mtb*, WT or ΔESX-1 (MOI = 1). At 3 dpi, phagosomal rupture was monitored in CD11b^+^ cells. (B, C) The blue CCF-4 signal overlays (B) and MFI_447 nm_ (C) are plotted for different Raw264.7 cell lines infected with *Mtb* WT. ** = statistically significant, as determined by the Student's *t* test, *p*<0.001. (D) Mycobacterial loads in different Raw264.7 cell lines, infected with various MOI of WT *Mtb*, as determined at 3 dpi. (E-G) Raw264.7::*nramp-1R* were transfected with Nramp-1-specific or scramble siRNA and the effective gene silencing was checked 3 days later by qRT-PCR (E). The siRNA-treated Raw264.7::*nramp*-1R cells were then infected with WT *Mtb* (MOI = 1) and studied for phagosomal rupture at 3 dpi. The blue CCF-4 signal (F) and the MFI_447 nm_ (G) are plotted. The results are representative of at least 3 experiments.

We next evaluated whether the characteristic *Mtb*-mediated partial inhibition of phagosome acidification was connected to the phenomenon of phagosome rupture. Given the previously established role of Natural resistance-associated macrophage protein (Nramp)-1, a phagosomal bivalent cation transporter, in phagosomal acidification and pH regulation [47–49], we evaluated its possible impact on mycobacteria-mediated phagosomal rupture. We thus used *Mtb* WT or ΔESX-1 strains to infect cells from the murine MΦ cell line Raw264.7, deficient in functional Nramp-1, which had been transfected with a non-functional *nramp-1S* (*Sensitive*) or a functional *nramp-1R* (*Resistance*) allele [50]. At 3 dpi, intense CCF-4 blue shifts were observed in WT *Mtb*-infected parental Raw264.7 cells and Raw264.7::Nramp-1S cells, whereas much less FRET inhibition was detected in Raw264.7::Nramp-1R cells (Fig. 4A-C). As assessed for various MOI, the intracellular mycobacterial load inside parental, Nramp-1S- or Nramp-1R-transfected Raw264.7 cells was comparable at 3 dpi, when the phagosomal rupture was monitored (Fig. 4D). Thus, the functional Nramp-1R seems to provide protection against *Mtb*-induced phagosomal rupture for the benefit of the host cell. The Nramp-1-mediated rescue of the host cells occurred at any MOI and independently of the host cell proliferation rate, which as we noticed, both influence the control of the infection (S5 Fig.). We obtained further confirmation of our results by using an *nramp-1* gene silencing strategy in Raw264.7::Nramp-1R cells (Fig. 4E), which reversed the phenotype and promoted *Mtb*-mediated phagosomal rupture (Fig. 4F-G).

**Fig 5:**
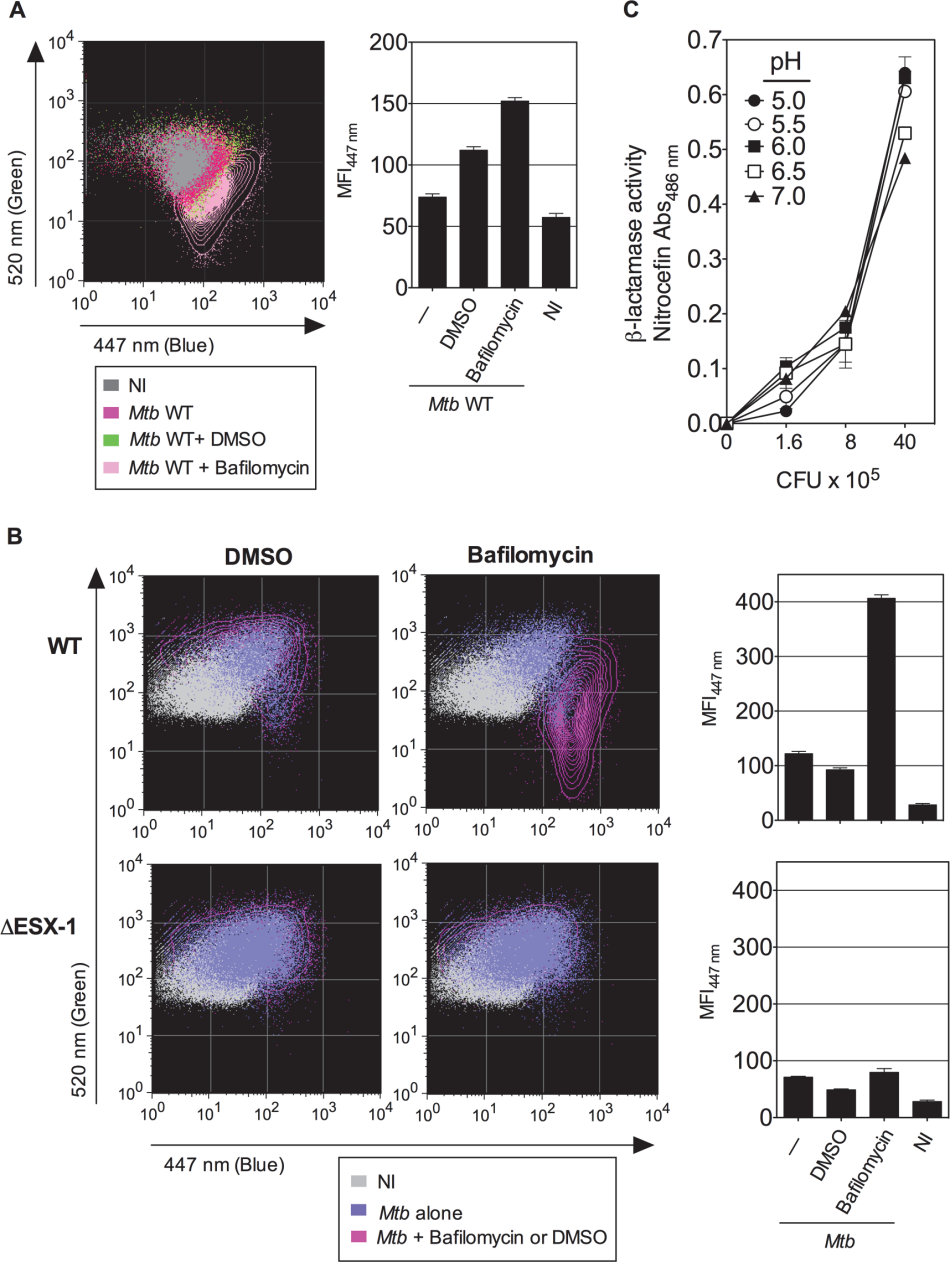
Inhibition of phagosomal acidification intensifies phagosomal rupture in *Mtb*-infected phagocytes. (A-B) Raw264.7::*nramp-1R* cells (A) or BM-DC from Sv129 *nramp-1R* mice (B) were treated with 20 nM of bafilomycin or DMSO 1h before infection with WT or ΔESX-1 *Mtb* (MOI = 1) and were assessed for phagosomal rupture at 4 dpi. (C) The intrinsic β-lactamase activity of WT *Mtb*, grown in Dubos broth with various pH, as measured by nitocephin, a chromogenic β-lactamase substrate. The results are representative of 2 experiments.

We further treated Raw264.7::Nramp-1R cells or, as primary phagocytes, BM-DC from Sv129 (*nramp-1R*) mice with bafilomycin, a specific inhibitor of vacuolar proton ATPases, prior to infection with WT *Mtb* H37Rv. As shown in Fig. 5A-B, the bafilomycin-mediated reduction of phagosomal acidification resulted in enhanced phagosomal rupture in both cell types. This observation provides additional evidence for a link between restriction of phagosome acidification and the strength of observed phagosomal rupture. In this FRET-based method, the β-lactamase operates on CCF-4 located in the host cytosol, where the pH remains neutral [25,41]. However, to further ascertain that the micro-environmental acidity did not affect the functionality of mycobacterial BlaC, we tested the β-lactamase enzymatic activity of *Mtb* at different pH levels by the use of nitrocefin, a chromogenic β-lactamase substrate. These experiments confirmed that *Mtb*, grown at different pH, ranging from 5 to 7, preserves entirely its β-lactamase enzymatic activity (Fig. 5C).

Thus, acidification of the phagosomal lumen seems to be a critical host cell parameters, which exerts an antagonistic effect on *Mtb*-mediated phagosomal rupture in phagocytes. The finding that both phenomena are linked provides a new basis for elucidating the molecular key players that govern the host-pathogen interaction during *Mtb* infection.

### ESX-1-dependent *Mtb*-mediated phagosome disruption in pulmonary phagocytes and *in vivo* in lungs and spleen of infected mice

**Fig 6:**
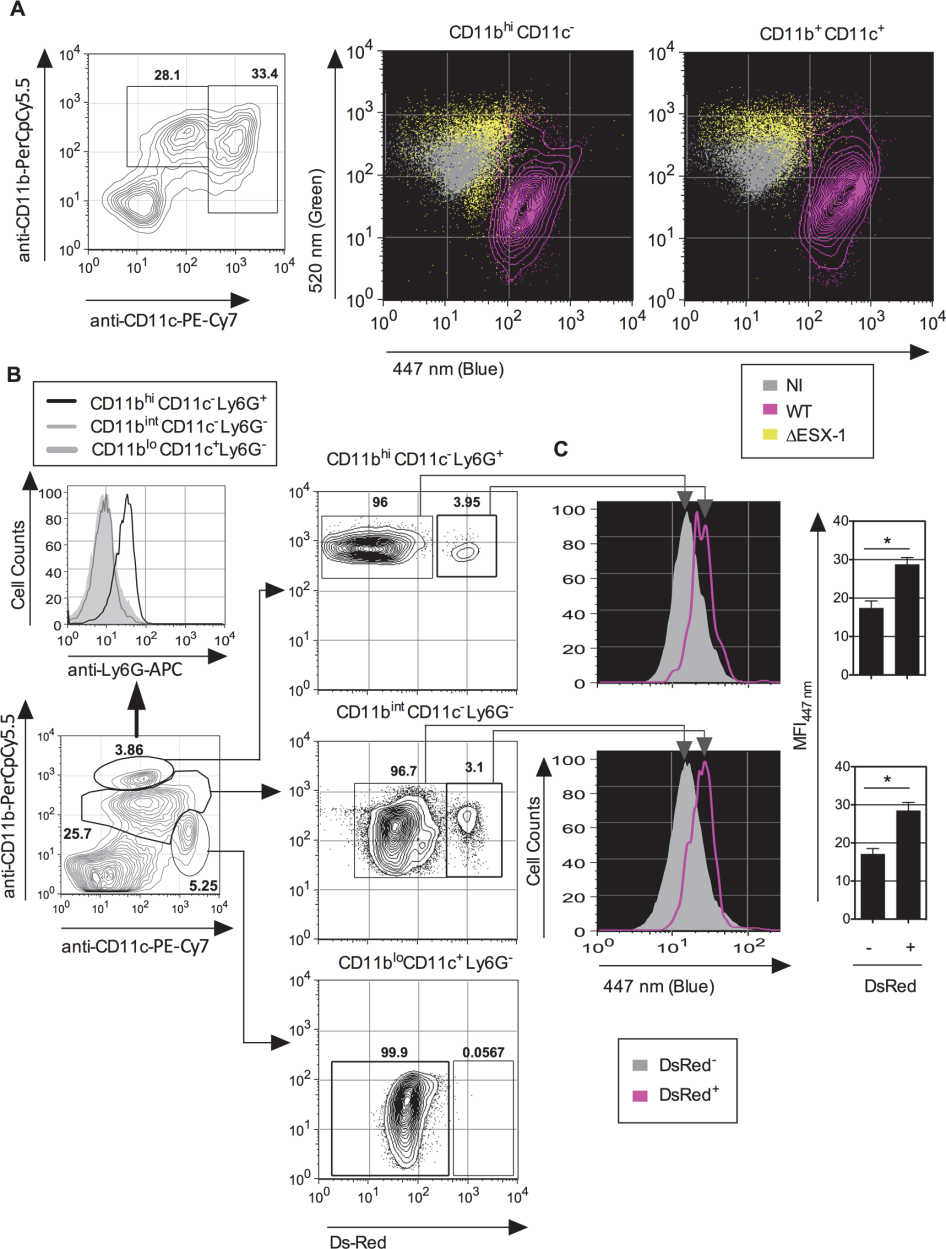
*Mtb*-mediated phagosomal rupture in different cell subsets *ex vivo* and *in vivo*. (A) Low-density cells were isolated from C57BL/6 mouse lung parenchyma and infected *ex vivo* with WT or ΔESX-1 *Mtb* (MOI = 1). Monocytes/MΦ (CD11b^+^ CD11c^-^) and DC (CD11b^int^ CD11c^+^) were assessed for CCF-4 signals at 4 dpi. (B) Phagosomal rupture detected *in vivo* in different *Mtb*-infected phagocyte subsets. C57BL/6 mice (*n* = 3) were injected i.v. with 1 x 10^6^ CFU of DsRed WT *Mtb* and at 3 weeks post infection. Alive low-density cells were isolated on Optiprep gradient from the spleen and were sequentially stained with CCF-4 and a cocktail of mAbs to distinguish neutrophils (CD11b^hi^ CD11c^-^ Ly6G^+^), MΦ/monocytes (CD11b^int^ CD11c^-^ Ly6G^-^) or DC (CD11b^lo^ CD11c^+^ Ly6G^-^). (C) Inside each innate cell subsets, the blue CCF-4 signals of the DsRed^+^ and DsRed^-^ cells were compared together. The results are representative of 2 independent experiments.

Previous studies on vacuolar rupture and phagosomal escape of *M*. *marinum* [12,51] and *Mtb* [13,15,16] used infected MΦ or DC under *in vitro* conditions. To extend our investigations towards cells from the lung, we examined the *Mtb*-mediated phagosomal rupture in different phagocyte types of mouse airways. To this end, low-density cells isolated from mouse lung parenchyma were infected *ex vivo* at an MOI of 1 with ΔESX-1 or WT *Mtb* strains. CCF-4 signals obtained from monocytes/MΦ (CD11b^hi^ CD11c^-^) and DC (CD11b^int^ CD11c^+^) were analyzed at 4 dpi, when changes in the FRET signal were detected in lung monocytes/MΦ and DC (Fig. 6A), showing the occurrence of *Mtb*-mediated phagosomal rupture in the primary lung phagocytes.

To assess the relevance of mycobacteria-mediated phagosomal rupture in phagocytes *in vivo*, in a first attempt we used T-/B-cell deficient *recombination activation gene* (*rag*) *2* knock-out mice in which infection with *Mtb* is more persistent and the innate cell compartments more developed than in their immunocompetent counterparts. However, flow cytometric analysis of lung- or spleen-derived MΦ/monocytes, DC and neutrophils obtained from infected (1 x 10^6^ CFU i.v. /mouse of WT or ΔESX-1 *Mtb*) or uninfected *rag2* ^*°/°*^ mice displayed indistinguishable CCF-4 blue profiles (S6 Fig.). The apparent failure in the detection of phagosomal rupture in this experimental setting seems to be related to the very low frequencies of mycobacteria-infected cells within each innate cell subset and/or a possible furtive feature of the phenomenon *in vivo* due to possible efferocytosis [52] of the primary phagocytes, in which phagosomal rupture and certain damage signals would have been initiated.

**Fig 7:**
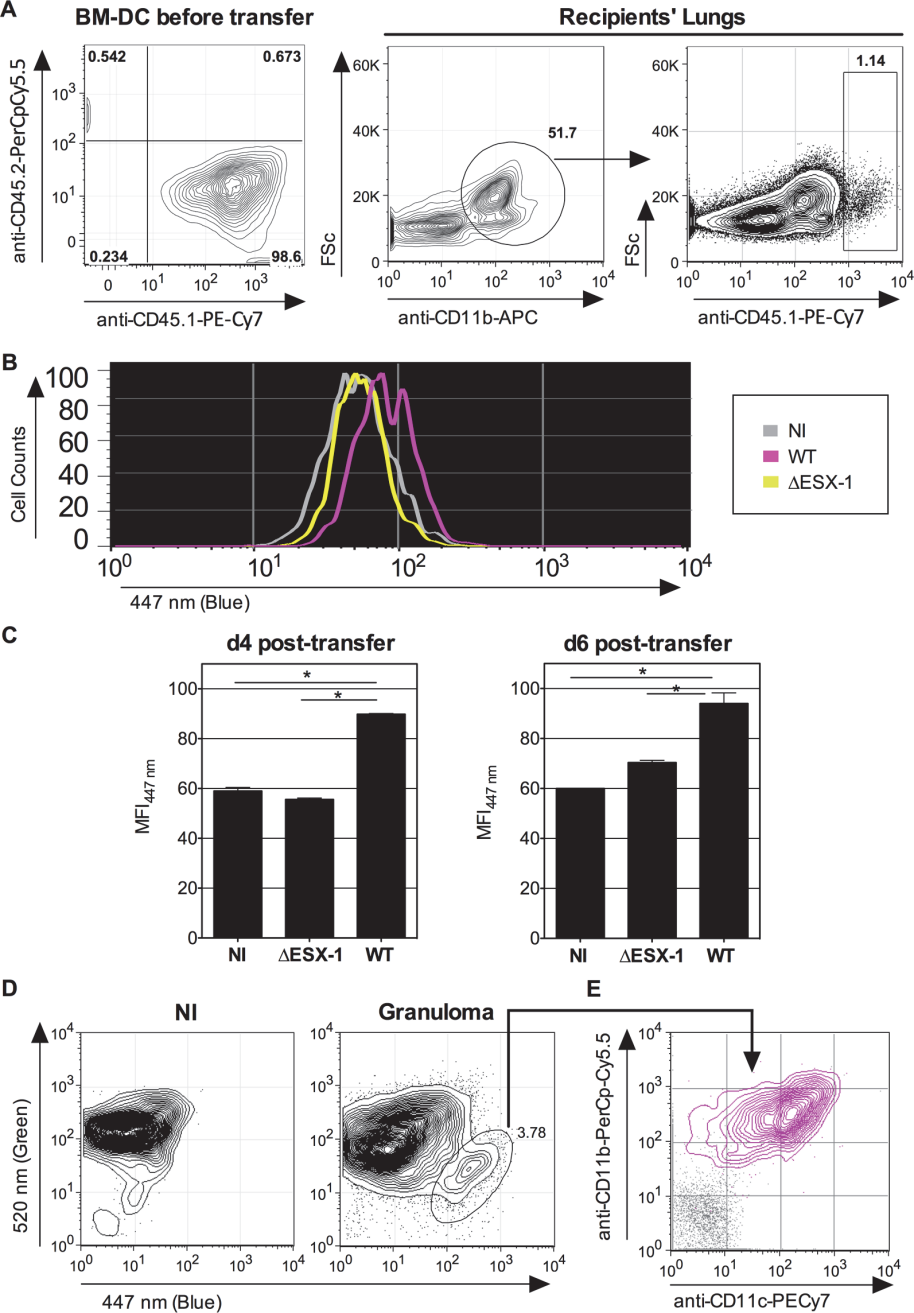
Detection of phagosomal rupture *in vivo* in *Mtb*-infected phagocytes. (A-B) BM-DC from CD45.1 donors were left non-infected or were infected with *Mtb* WT or ΔESX-1 (MOI = 1) for 16h. Of note, cultures of CD45.1 BM-DC, infected with DsRed-mycobacteria in the same conditions, showed that >70% of cells had uptaken mycobacteria like shown in Fig. 2A. Cells were recovered and transferred i.n. (2 x 10^6^ cells/mouse) into CD45.2 congenic recipients. (B) Four days post-transfer, alive lung low-density cells from the recipients (*n* = 3/group) were isolated on Optiprep gradient and incubated with mAbs specific to CD11b and CD45.1, subsequent to incubation with CCF-4. The blue signals from the three experimental groups are overlaid. (C) Histograms show the comparative blue CCF-4 signals in the CD11b^+^ CD45.1^+^ cells from different groups at days 4 or 6 post transfer. * = statistically significant, as determined by the Student's *t* test, *p*<0.01. (D) Lung granuloma from C57BL/6 mice, infected via aerosol route with ≈200 CFU/mouse of WT *Mtb*, were removed at 6 weeks p.i. and were treated with collagenase and DNAse-I and enriched in low-density cells. In parallel to these cells, lung low-density cells from uninfected controls were assessed for CCF-4 blue switch. (E) CD11b *vs*. CD11c surface expression of the cells showing the increased CCF-4 shift (pink), compared to unstained negative control incubated with control Ig isotypes (gray). The results are representative of 2 independent experiments.

To distinguish infected and non-infected cells, we then used fluorescent DsRed-WT *Mtb* (1 x 10^6^ CFU/mouse) for intravenous (i.v.) infection of C57BL/6 mice, which allowed us to focus on the relatively few *Mtb*-infected phagocytes present during the initial phase of chronic infection. At 3 weeks p.i. mice were sacrificed, the spleens homogenized and resulting cells enriched and subjected to flow cytometric analysis. We have focused on the phagocytes of the spleen because this organ is particularly targeted by the i.v. route of infection. When the CCF-4 blue signal of the innate immune cells that contained DsRed *Mtb* was compared to the other cells inside each cell subset in the spleen (Fig. 6B), a slight increase in CCF-4 blue signal was notably detected in *Mtb*-containing cells in the subsets of neutrophils (CD11b^hi^CD11c^-^Ly6G^+^) and MΦ/monocytes (CD11b^int^CD11c^-^Ly6G^-^) (Fig. 6C), which suggests the occurrence of weak, albeit reproducible, levels of phagosomal rupture in these infected cells. Interestingly, no DsRed^+^ cells were detected inside the CD11b^lo^CD11c^+^Ly6G^-^ DC subset, which might be due to possible rapid turnover of infected DC or to their CD11b up-regulation. In this chronic infection model, it was however not possible to compare WT and ΔESX-1 *Mtb* strains, because of the non-persistence of the latter. To overcome this limitation we developed an alternative *in vivo* model whereby mice were instilled intra-nasally with cells that were infected with *Mtb in vitro* prior to transfer, and whose infection status *in vivo* could be specifically monitored. To this end, BM-DC from mice with CD45.1 hematopoietic allelic marker were infected *in vitro* with WT or ΔESX-1 *Mtb*, in conditions that allowed up to 70% of the cells to be infected (Fig. 2A), whereas control cells were left uninfected. At 16 hpi, the cells were instilled into the airways of congenic CD45.2 recipients. At different time points post-transfer, the lung low-density cells were isolated and the CCF-4 blue shift in the CD11b^+^ CD45.1 cell subset of the different experimental groups assessed (Figs. 7A and S7). Strikingly, at day 4 and day 6 post-transfer, in the CD11b^+^ CD45.1 population infected with WT *Mtb*, a blue shift was detected in comparison to the non-infected or ΔESX-1-infected transferred cells (Fig. 7B-C).

Moreover, independent flow cytometric examination of cells extracted directly from surface lung granuloma tissue of *Mtb*-infected C57BL/6 mice revealed a small, distinct cell population that displayed a clear-cut blue signal and a CD11b^+^ CD11c^+^ phenotype (Fig. 7D), which points to the presence of innate cells in these lungs wherein *Mtb*-mediated phagosomal rupture had occurred.

Altogether, our data suggest that the *Mtb*-induced phagosomal rupture does indeed happen *in vivo*, in *Mtb*-infected cells in the organs of small laboratory animals. The detected phagocytes containing intracellular bacteria seem to have a life-time of several days, which however does not exclude the possibility that a portion of the total number of infected phagocytes might get eliminated by efferocytosis [52], as suggested by the relatively modest differences in blue shift observed in the *in vivo* settings.

## Discussion

The pathogenic potential of *Mtb* is intimately linked to the interplay between the host defense and the persistence of the mycobacteria. The intracellular localization and cytosolic access of the bacterium has substantial consequences on the recognition of mycobacteria-associated patterns by the cytosolic receptors of the innate immunity that determine innate and adaptive immune responses and ultimately the fate of the host cell and the bacterium [27]. Subsequent to phagocytosis, in order to avoid the acidified environment generated by the phagosome-lysosome fusion, some specialized intracellular bacteria, such as *S*. *flexneri*, *Listeria monocytogenes* or *Francisella tularensis*, evolved to rapidly escape from phagosomes into the cytosol [21,53,54]. In contrast, *Mtb* has been described as a bacterium that resists degradation in the phagosome by inhibiting the fusion with lysosomes, a characteristic feature that seems to protect the bacilli from bactericidal mechanisms of the phagocytes and allows intracellular survival and multiplication [10,18,55–57]. However, recent reports based on *in vitro* infection of phagocytes also suggest that at later stages of infection ESX-1-dependent vacuolar breakage might be an important requirement for the pathogenic potential of *Mtb*, given that ESX-1-deficient bacilli that are unable to perforate and lyse the phagosomal membrane are—in general—attenuated [13,15,16,18,56–59].

In previous studies, *Mtb*-mediated phagosomal escape has only been reported at late time points like 2–5 dpi, a kinetic feature that was not reconcilable with the intracellular host immune events, like type I IFN induction, which require the early recognition of mycobacterial components by cytosolic sensors. Here, the use of highly sensitive FRET-based cytometry enabled us to highlight minor levels of cytosolic contact of *Mtb* and its products initiated as soon as 3 hpi, which is kinetically concordant and proportional with the amounts of IFN-β released by DC. While we cannot exclude the possibility that some of this effect may have been caused by bacterial products translocating through permeable phagosomal membranes [30], the reproducible differences observed between the WT and the ΔESX-1 *Mtb* strains argue for a specific, ESX-1-mediated impact. We also noted that distinct cell types might display different susceptibility to phagosomal rupture, with THP-1 cells as the most susceptible ones, followed by BM-DC/BM-MΦ, and the Raw264.7 MΦ as the least affected cell types, tested.

Our results show that the phagosomal bivalent cation transporter Nramp-1 interferes with *Mtb*-induced phagosomal rupture as observed at 3 dpi, i.e., a time point at which mycobacterial loads were still comparable in *Mtb*-infected MΦ harboring Nramp-1S (non functional)- or Nramp-1R (functional) allelic forms. In line with that, the effect of bafilomycin, reported to inhibit phagosomal acidification [60], reconstituted in Nramp-1R-proficient phagocytes the capacity of *Mtb* to enhance phagosomal rupture to the level of Nramp-1S phagocytes. Thus, the partial inhibition of phagosome acidification emerges as a prerequisite to mycobacterial phagosomal rupture. Plausibly, only when phagosome acidification is partially inhibited, mycobacteria may survive, use their virulence factors and induce phagosomal membrane disruption.

Although cellular models may provide important new insights into cell biological mechanisms, evaluation of the accuracy of the findings in an *in vivo* model, i.e. in tissues or organs is of crucial importance to emphasize their relevance. Previous electron microscopy analyses of lung innate cells isolated from TB patients or mycobacteria-infected mice have led to discrepancies with regards to intracellular location [18]. In alveolar MΦ of TB patients and in granuloma or lung homogenates of infected mice, *Mtb* has been detected as single bacterium or pairs of bacilli inside phagosomes [61,62], whereas *Mtb* has also been observed in membrane-disrupted compartments or free in the cytosol in the mouse granulomas [63,64]. Moreover, heavily infected human alveolar MΦ [62] and damaged mouse MΦ of inflammatory sites [65] contain multiple mycobacteria per phagosome. In this context, our results from carefully designed *in vivo* infection experiments add new elements to the discussion. Although the strength of the FRET-inhibition was found weaker under *in vivo* conditions (Figs. 6 and 7) than observed for the cell culture-based infection assays (Figs. 1 and 2), the reproducibility and complementarity of the results from the three distinct *in vivo* settings analyzed, point to biological relevance of mycobacteria-induced phagosomal rupture in the organs of *Mtb*-infected laboratory animals. It should be noted that in our experiment with BM-DC from mice with the CD45.1 hematopoietic allelic markers (Fig. 7), we cannot exclude that in the infected DC some minor cytosolic contact might develop already *in vitro*, prior to their instillation to the CD45.2 recipient mice. However, the finding that FRET inhibition remains detectable for several days after the transfer into the lungs of the CD45.2 recipients suggests that the phagocytes in which cytosolic access of *Mtb* progressively builds up, can survive in the host environment for some days. Together with *ex vivo* results from MΦ/monocytes and DC isolated from the lung parenchyma, the *in vivo* demonstration of cytosolic access of *Mtb* provides important new insights into the cellular events during infection inside the organs. Our data suggest that after infection, the concerned phagocytes may persist in the organs long enough to have a potential impact on host defense mechanisms that likely also include key cellular processes, such as autophagy, which requires *Mtb* ubiquitination in an ESX-1-dependent manner [16,33,38,39].

The intracellular localization of mycobacteria and mycobacteria-mediated phagosomal rupture have been subject of numerous controversies, which may be explained by the differences between the level of virulence of mycobacterial strains used, the MOI and the conditions of the mycobacterial cultures *in vitro* [18]. For the virulent strains, here we used WT and DsRed *Mtb* previously passaged in immunocompetent mice to maintain a normal degree of virulence and to remain as close to natural infection as possible. We only used mycobacterial cultures in mid-log_10_ growth phase to minimize bacterial mortality, and we cultured the bacteria in the presence of Tween 80 to avoid clumping, as phagocytosis of non-viable or clumped mycobacteria may lead to rapid phagosome-lysosome fusion and prevent visualization of phagosomal rupture [18]. In addition, we systematically compared the ESX-1-proficient and ESX-1-deficient mycobacterial strains and detected a relevant phagosomal rupture only with ESX-1-proficient strains.

Previous observations with numerous virulent and attenuated *Mtb* strains suggest that the capacity of a strain to induce phagosomal rupture *in vitro* is often correlated with its virulence [15,16]. Hence, the ESX-1-dependent, mycobacteria-induced phagosomal rupture emerges as a major characteristic feature of *Mtb* infection, which likely initiates the first damages caused by this intracellular pathogen to the host cell. Consequently, modulation of the parameters, which orchestrate this phenomenon, may constitute a promising base for vaccinal or therapeutic interventions against TB. For example, we have previously noticed that recombinant BCG and *M*. *microti* strains with a reconstituted ESX-1 secretion system showed enhanced protective efficacy [66,67]. More recently, a dedicated study identified small molecule inhibitors belonging to the benzyloxybenzylidene-hydrazine and the benzothiophene chemical classes, which interfered with ESAT-6 secretion and thereby protected host cells from *Mtb*-induced lysis [68]. Molecules belonging to closely related chemical scaffolds were also identified in a high content phenotypic screen as agents that interfered with the intracellular growth and the virulence of *Mtb* [69]. Hence, it is conceivable that future phenotypic library screening might identify novel pharmacological compounds that inhibit *Mtb*-mediated phagosomal rupture in the host cell. Such molecules would represent interesting anti-virulence compounds to be tested as addition to conventional treatment regimens against TB.

In conclusion, our study suggests that *Mtb* is not the passive pathogen that induces pathology only by the over-boarding reaction of the host immune system. We show that ESX-1-mediated phagosomal rupture contributes in a significant way to establish mycobacterial cytosolic contact, which is however only possible if the maturation / acidification of the phagosome is limited in a first process. In this direction, our study also opens new perspectives for future studies on the mycobacterial components involved in the modulation of phagosomal acidification such as the phthiocerol dimycocerosates and other mycobacterial factors, reported to intervene in this process [70,71].

The ESX-1 system might thus represent one of the final members in a chain of virulence factors that determine the pathogenicity of *Mtb* through the induction of phagosomal rupture, and its function might therefore have been evolutionary preserved [5,7]. As such, our work has the potential to reconcile the outcome of previous studies on mycobacterial virulence factors that interfere with vacuolar acidification [71–74] and studies on cellular localization of *Mtb* [13–16] and establishes *Mtb*-mediated phagosomal rupture as a basic biological mechanism involved in TB pathogenesis.

## Materials and Methods

### Animal infection model

C57BL/6 mice, *rag2* ^°/°^ or CD45.1 were obtained from Animal Facilities of Institut Pasteur. C57BL/6 mice were purchased from Janvier Le Genest-Saint-Isle France). CD45.2 mice were anesthetized by i.p. injection of 100 mg/kg Ketamine (Lyon, France) and 10 mg/kg Xylazine (KCP Kiel, Germany) before cell transfer by i.n. route. Mouse infection with *Mtb* via aerosol route was performed as previously described [75]. Granuloma were recovered from the surface lung parenchyma of infected C57BL/6 mice at 6 weeks p.i.

### Ethics statement

Mouse studies were approved by the Institut Pasteur Safety Committee, in accordance with French and European guidelines and regulations (Directive 86/609/CEE and Decree 87–848 of 19 October 1987) and the Animal Experimentation Ethics Committee Ile-de-France-1 (reference number 2012–0005).

### Cell cultures

THP-1 cells (our laboratory stock collection, initially originating from ATCC provided cells) were maintained in RPMI, complemented with 10% heat-inactivated FBS and were treated with 20 ng/ml of Phorbol 12-Myristate 13-Acetate for 72h to induce their differentiation into MΦ. Raw264.7 cells transfected with *nramp-1S* or -*1R* allele (kind gift of Pr J. Blackwell) [50] were treated with 8 μg/ml of the selective antibiotic puromycin.

BM-MΦ or -DC were generated from femur hematopoietic precursors, respectively by use of M-CSF or GM-CSF. Rat anti-mouse IFN-α mAb (RMMA-1), biotinylated polyclonal rabbit anti-mouse IFN-α (R&D), rat anti-mouse IFN-β (8.S.415) (LifeSpan BioSciences) and biotinylated polyclonal rabbit anti-mouse IFN-β (R&D) were used to quantify the cytokines produced in the culture supernatants by ELISA.

### *Mtb* cultures and cell infection

*Mtb* H37Rv, WT, ΔESX-1 (kind gift of Pr. W. Jacobs) [10] or ΔESX::ESX-1 [42] were maintained in 7H9 medium supplemented with ADC (Difco). Seven-to-10 days before cell infection, bacteria were transferred into Dubos medium, which contains Tween 80, to avoid mycobacterial clumping. DsRed-WT or -ΔESX-1 strains were obtained by complementation with the pMRF plasmid containing a DsRed cassette, under the hsp60 promoter (kind gift of Dr. S. Cho) and were cultured in the continuous presence of 20 μg/ml of the selective antibiotic kanamycin. In *in vivo* experiments, we used an *Mtb* H37Rv strain with a plasmid containing the DsRed and hygromycin resistance genes (kind gift of Dr. O. Neyrolles). Only mycobacteria grown to mid-log_10_ phase were used to minimize the frequency of death bacteria.

Raw264.7 cells were infected at various MOI with *Mtb* in complete antibiotic-free RPMI. At 3 dpi, equal numbers of cells were lysed by addition of 0.1% Triton X-100 in PBS and the intracellular CFU was determined by plating serial dilutions of cell lysates on 7H9 Agar medium and incubation at 37°C for 3 weeks.

### CCF-4 assay and flow cytometry

The principle of the β-lactamase CCF-4 FRET assay is summarized in S1 Fig.. To measure the *Mtb* phagosomal rupture, cells were stained during 1h at RT, with 8 μM CCF-4 (Invitrogen) in EM buffer (120 mM NaCl, 7 mM KCl, 1.8 mM CaCl_2_, 0.8 mM MgCl_2_, 5 mM glucose and 25 mM Hepes, pH 7.3) complemented with 2.5 μM probenecid. Cells were then stained with anti-CD11c-PE-Cy7, anti-CD11b-PerCp-Cy5.5 (eBiosciences) or anti-CD11b-APC (BD) mAbs andfixed with 4% PFA overnight at 4°C. Cell mortality in the same cultures of infected cells was determined by use of Pacific Blue Dead/Live reagent (Invitrogen), which reacts with free amines both inside and outside of the plasma membrane, yielding log_10_ 1 more intense fluorescent staining of dead cells. Anti-CD45.1-PE-Cy7 and anti-CD45.2-PerCpCy5.5 were from eBiosciences. To avoid fluorochromes with emission signals overlapping with those of CCF-4 (λ_em_ 500–550 nm and λ_em_ 410–470 nm), APC (λ_em_ 660 nm)-, PerCp-Cy5.5 (λ_em_ 696 nm)- or PE-Cy7 (λ_em_ 778 nm)-conjugated mAbs were chosen for concomitant cell surface staining. Cells were analyzed in a CyAn cytometer using Summit software (Beckman Coulter, France). At least 100,000 events per sample were acquired for *in vitro* assays. For *in vivo* detection of CCF-4 signal in CD45 congenic mouse model, 1,000,000 events per sample have been acquired. Data were analyzed with FlowJo software (Treestar, OR).

### Gene silencing

siRNA transfection to cells was performed by using reverse transfection method. A pool of four Nramp-1-specific siRNAs, GGUCAAGUCUAGAGAAGUA, GAUCCUAGGCUGUCUCUUU, GGGCGACUGUGCUAGGUUU and GAAGUCAUCGGGACGGCUA, at final concentration of 50 nM, was mixed with 6 μl of lipofectamine (Invitrogen) in 500 μl of PBS in 6-well plates. After 30 min incubation at RT, 3 x 10^5^ cells contained in 2 ml of complete RPMI were added to the mixture and incubated for 3 days at 37°C. The efficiency of gene silencing was determined by qRT-PCR before the infection. One mg of total RNA was transcribed into cDNA. Then, 4 μL of cDNA was tested by qRT-PCR with LightCycler 480 SYBR Green using GCCACTGTGCTAGGTTTGCT and AATGGTGATCAGTACACCGC primers. All experiments were run in triplicate and the Livak method [76] was applied for relative quantification with β-actin.

### Mycobacterial β-lactamase activity assay

The β-lactamase activity of *Mtb*, grown in Dubos broth with various pH, was measured by use of the chromogenic β-lactamase substrate, nitrocefin. Briefly 1 x 10^6^ bacteria, re-suspended in 100 μl of Dubos broth at indicated pH, were incubated in 96-well plates with 50 μl of nitrocefin, reconstituted at 0.5 mg/ml in PBS which contained 5% DMSO. Absorbance by nitrocefin at 486 nm was measured after 3 hours of incubation at 37°C.

### Enrichment of innate immune cells

Lungs or spleen were removed aseptically and were digested by treatment with 400 U/ml type IV collagenase and DNase I (Roche). Following a 45 min incubation at 37°C, single-cell suspensions were prepared by use of a Gentle Macs (Miltenyi) and by passage through 100-μm nylon filters (Cell Strainer, BD Falcon). When indicated, cell suspensions were enriched in low-density cells on iodixanol gradient medium (OptiPrep, Axis-Shield), according to the manufacturer’s protocol. Notably this gradient only selects alive cells, as confirmed by blue Trypan exclusion assay. These cells were either used directly in flow cytometry analyses or were plated in 12 well culture plates in complete RPMI to be infected *ex vivo* with mycobacteria.

## Supporting Information

S1 Fig Cartoon of the principle of the CCF-4 based FRET assay. In step 1, CCF4-AM (Life Technologies) represents a lipophilic, esterified form of the CCF4 substrate, which allows it to readily enter cells. As shown for step 2, upon entry, cleavage by endogenous cytoplasmic esterases rapidly converts CCF4-AM into its negatively charged form, CCF4, which is retained in the cytosol and thus cannot enter into the different cell organelles, including phagosomes containing bacteria. In case the bacteria remain engulfed in the intact phagosome, the endogenous bacterial beta-lactamase can not reach the CCF-4 substrate and upon stimulation at ~ 409 nm, CCF-4 shows Fluorescence Resonance Energy Transfer (FRET) that leads to emmision of “green” light at ~ 535 nm. As shown for step 3, in case of phagosomal rupture and cytosolic contact the mycobacterial β-lactamase (shown as red dots linked to the bacterium) gets in contact with the CCF-4 substrate trapped in the cytosol, and is inducing cleavage of the substrate and inhibiting FRET, thereby leading to emission of “blue” light at ~ 450 nm.
(JPG)

S2 Fig Effect of PFA fixation on CCF-4 blue shift and effect of complementation of ΔESX-1 H37Rv mutant with complete ESX-1 genomic region on the capacity of *Mtb* to induce phagosomal rupture. PFA fixation of mycobacteria-infected cells results in some levels of CCF-4 bleu shift. (A) PFA fixation of 1 cells or (B) BM-DC infected with *M*. *bovis* BCG, deficient in ESX-1, results in low CCF-4 shift to blue, which is plausibly linked to a small leak of β-lactamase activity into the cytosol soon after the cell fixation prior to signal acquisition. However, these levels of shift are ten to hundred of times lower compared to those observed with cells infected with ESX-1-sufficient mycobacteria. (C) Complementation of ΔESX-1 H37Rv mutant with complete ESX-1 genomic region restores the capacity of *Mtb* to induce phagosomal rupture. Phagosomal rupture induced by WT, ΔESX-1 or ΔESX-1 complemented with complete ESX-1-region in infected BM-DC (MOI = 1), as determined by the profile of green *vs*. blue CCF-4 signals at 5 dpi. MFI_447 nm_ values in different infected BM-DC groups are indicated.
(JPG)

S3 Fig Progressive phagosomal rupture assessed in DC infected with DsRed-WT *Mtb*. Cultures of BM-DC were infected with DsRed-WT *Mtb* (MOI = 1) and the cells were analyzed from 3 to 5 dpi. (A) Cells containing DsRed *Mtb* were gated and (B) their CCF-4 blue signal were overlayed and compared to that of uninfected cells.
(JPG)

S4 Fig Early secretion of IL-1β by BM-DC subsequent to *Mtb* infection. IL-1β concentrations, as quantified in the supernatants of BM-DC shown in Fig. 3, at 3, 24 and 48 h following infection with *Mtb* WT or ΔESX-1 at different MOI.
(JPG)

S5 Fig Nramp-1R confers resistance to phagosomal rupture subsequent to *Mtb* infection regardless of the MOI and the host cell proliferation. Effect of different FBS percentages in the culture medium, directly governing the rate of Raw246.7 cell proliferation and different MOI of WT or ΔESX-1 *Mtb*, as evaluated in Raw246.7 cells, parental or transfected with *nramp-1S* or *nramp-1R*. Shown are comparative blue CCF-4 signals. It is noteworthy that, compared to THP-1 cells, BM-DC or BM-MΦ, relatively low levels of phagosomal rupture were generally observable in Raw264.7 MΦ. Indeed, a CCF-4 blue shift is weakly detectable at 2 dpi, peaks at 3 dpi and then decreases as soon as 4 dpi. This feature seems to be linked to intense proliferative capacity of these cells despite the infection and also to their possible intense efferocytic capacity.
(JPG)

S6 Fig Attempt to detect *in vivo* phagosomal rupture in *Mtb*-infected phagocytes. T-/B-cell deficient *rag2* ^*°/°*^ mice were infected i.v. with 1 x 10^6^ CFU/mouse. At 1, 2 or 3 wks p.i., low density cells from the spleen were stained with CCF-4 and subsequently with cocktails of mAbs to distinguish different innate cell subsets, i.e., DC (CD11c^+^ CD11b^+^), MΦ/monocytes (CD11c^—^CD11b^+^) or neutrophils (CD11b^+^ Ly6G^+^). Shown are results obtained at 2 wks p.i.. Comparable results were obtained at 1 or 3 wks p.i. with both spleen and lung low density cells.
(JPG)

S7 Fig Gating strategy on CD45.1 donor innate cells adoptively transferred into the CD45.2 recipients. BM-DC from CD45.1 donor mice, non-infected or infected with *Mtb* ΔESX-1 or WT, were transferred i.n. into the CD45.2 recipients. Shown are the low-density cells recovered from the lung parenchyma of the recipients of each group at day 4 post transfer. Cells were first gated on FSc/CD11b and then for CD45.1^+^ cells. The comparative CCF-4 blue signal of such cells from different experimental groups are shown in Fig. 7C.
(JPG)

## References

[1] Abdallah AM, Gey van Pittius NC, Champion PA, Cox J, Luirink J, et al (2007) Type VII secretion system of mycobacteria show the way. Nat Rev Microbiol 5: 883–891. 17922044 10.1038/nrmicro1773

[2] Majlessi L, Prados-Rosales R, Casadevall A, Brosch R (2015) Release of mycobacterial antigens. Immunol Rev 264: (1–21; in press). 10.1111/imr.12251 25703550

[3] Bitter W, Houben EN, Bottai D, Brodin P, Brown EJ, et al (2009) Systematic genetic nomenclature for type VII secretion systems. PLoS Pathog 5: e1000507 10.1371/journal.ppat.1000507 19876390 PMC2763215

[4] Houben EN, Korotkov KV, Bitter W (2014) Take five—Type VII secretion systems of mycobacteria. Biochim Biophys Acta 1844: 1707–1716. 10.1016/j.bbamcr.2013.11.003 24263244

[5] Gonzalo-Asensio J, Malaga W, Pawlik A, Astarie-Dequeker C, Passemar C, et al (2014) Evolutionary history of tuberculosis shaped by conserved mutations in the PhoPR virulence regulator. Proc Natl Acad Sci U S A 111: 11491–11496. 10.1073/pnas.1406693111 25049399 PMC4128152

[6] Supply P, Marceau M, Mangenot S, Roche D, Rouanet C, et al (2013) Genomic analysis of smooth tubercle bacilli provides insights into ancestry and pathoadaptation of *Mycobacterium tuberculosis* . Nat Genet 45: 172–179. 10.1038/ng.2517 23291586 PMC3856870

[7] Boritsch EC, Supply P, Honore N, Seeman T, Stinear TP, et al (2014) A glimpse into the past and predictions for the future: the molecular evolution of the tuberculosis agent. Mol Microbiol 93: 835–852. 10.1111/mmi.12720 25039682

[8] Stinear TP, Seemann T, Harrison PF, Jenkin GA, Davies JK, et al (2008) Insights from the complete genome sequence of *Mycobacterium marinum* on the evolution of *Mycobacterium tuberculosis* . Genome Res 18: 729–741. 10.1101/gr.075069.107 18403782 PMC2336800

[9] de Jonge MI, Pehau-Arnaudet G, Fretz MM, Romain F, Bottai D, et al (2007) ESAT-6 from *Mycobacterium tuberculosis* dissociates from its putative chaperone CFP-10 under acidic conditions and exhibits membrane-lysing activity. J Bacteriol 189: 6028–6034. 17557817 10.1128/JB.00469-07 PMC1952024

[10] Hsu T, Hingley-Wilson SM, Chen B, Chen M, Dai AZ, et al (2003) The primary mechanism of attenuation of bacillus Calmette-Guerin is a loss of secreted lytic function required for invasion of lung interstitial tissue. Proc Natl Acad Sci U S A 100: 12420–12425. 14557547 10.1073/pnas.1635213100 PMC218773

[11] Smith J, Manoranjan J, Pan M, Bohsali A, Xu J, et al (2008) Evidence for pore formation in host cell membranes by ESX-1-secreted ESAT-6 and its role in *Mycobacterium marinum* escape from the vacuole. Infect Immun 76: 5478–5487. 10.1128/IAI.00614-08 18852239 PMC2583575

[12] Stamm LM, Morisaki JH, Gao LY, Jeng RL, McDonald KL, et al (2003) *Mycobacterium marinum* escapes from phagosomes and is propelled by actin-based motility. J Exp Med 198: 1361–1368. 14597736 10.1084/jem.20031072 PMC2194249

[13] van der Wel N, Hava D, Houben D, Fluitsma D, van Zon M, et al (2007) *M*. *tuberculosis* and *M*. *leprae* translocate from the phagolysosome to the cytosol in myeloid cells. Cell 129: 1287–1298. 17604718 10.1016/j.cell.2007.05.059

[14] Hagedorn M, Rohde KH, Russell DG, Soldati T (2009) Infection by tubercular mycobacteria is spread by nonlytic ejection from their amoeba hosts. Science 323: 1729–1733. 10.1126/science.1169381 19325115 PMC2770343

[15] Simeone R, Bobard A, Lippmann J, Bitter W, Majlessi L, et al (2012) Phagosomal rupture by *Mycobacterium tuberculosis* results in toxicity and host cell death. PLoS Pathog 8: e1002507 10.1371/journal.ppat.1002507 22319448 PMC3271072

[16] Houben D, Demangel C, van Ingen J, Perez J, Baldeon L, et al (2012) ESX-1-mediated translocation to the cytosol controls virulence of mycobacteria. Cell Microbiol 14: 1287–1298. 10.1111/j.1462-5822.2012.01799.x 22524898

[17] Fortune SM, Rubin EJ (2007) The complex relationship between mycobacteria and macrophages: it's not all bliss. Cell Host Microbe 2: 5–6. 18005712 10.1016/j.chom.2007.06.008

[18] Harriff MJ, Purdy GE, Lewinsohn DM (2012) Escape from the phagosome: The explanation for MHC-I processing of mycobacterial antigens? Front Immunol 3:40: 10.3389/fimmu.2012.00040 22566923 PMC3342008

[19] Molloy S (2012) BACTERIAL PATHOGENESIS TB blurs the lines. Nature Reviews Microbiology 10: 442–442. 10.1038/nrmicro2825 22699958

[20] Friedrich N, Hagedorn M, Soldati-Favre D, Soldati T (2012) Prison break: pathogens' strategies to egress from host cells. Microbiol Mol Biol Rev 76: 707–720. 10.1128/MMBR.00024-12 23204363 PMC3510522

[21] Ray K, Bobard A, Danckaert A, Paz-Haftel I, Clair C, et al (2010) Tracking the dynamic interplay between bacterial and host factors during pathogen-induced vacuole rupture in real time. Cell Microbiol 12: 545–556. 10.1111/j.1462-5822.2010.01428.x 20070313

[22] Flores AR, Parsons LM, Pavelka MS Jr. (2005) Genetic analysis of the beta-lactamases of *Mycobacterium tuberculosis* and *Mycobacterium smegmatis* and susceptibility to beta-lactam antibiotics. Microbiology 151: 521–532. 15699201 10.1099/mic.0.27629-0

[23] Malen H, Pathak S, Softeland T, de Souza GA, Wiker HG (2010) Definition of novel cell envelope associated proteins in Triton X-114 extracts of *Mycobacterium tuberculosis* H37Rv. BMC Microbiol 10:132 10.1186/1471-2180-10-132 20429878 PMC2874799

[24] Charpentier X, Oswald E (2004) Identification of the secretion and translocation domain of the enteropathogenic and enterohemorrhagic Escherichia coli effector Cif, using TEM-1 beta-lactamase as a new fluorescence-based reporter. J Bacteriol 186: 5486–5495. 15292151 10.1128/JB.186.16.5486-5495.2004 PMC490934

[25] Nothelfer K, Dias Rodrigues C, Bobard A, Phalipon A, Enninga J (2011) Monitoring *Shigella flexneri* vacuolar escape by flow cytometry. Virulence 2: 54–57. 10.4161/viru.2.1.14666 21317555

[26] Majlessi L, Brodin P, Brosch R, Rojas MJ, Khun H, et al (2005) Influence of ESAT-6 secretion system 1 (RD1) of *Mycobacterium tuberculosis* on the interaction between mycobacteria and the host immune system. J Immunol 174: 3570–3579. 15749894 10.4049/jimmunol.174.6.3570

[27] Behar SM, Divangahi M, Remold HG (2010) Evasion of innate immunity by *Mycobacterium tuberculosis*: is death an exit strategy? Nat Rev Microbiol 8: 668–674. 10.1038/nrmicro2387 20676146 PMC3221965

[28] Behar SM, Martin CJ, Booty MG, Nishimura T, Zhao X, et al (2011) Apoptosis is an innate defense function of macrophages against *Mycobacterium tuberculosis* . Mucosal Immunol 4: 279–287. 10.1038/mi.2011.3 21307848 PMC3155700

[29] Aguilo J, Alonso H, Uranga S, Marinova D, Arbues A, et al (2013) ESX-1-induced apoptosis is involved in cell-to-cell spread of *Mycobacterium tuberculosis* . Cell Microbiol. 15:1994–2005. 10.1111/cmi.12169 23848406

[30] Nakamura N, Lill JR, Phung Q, Jiang Z, Bakalarski C, et al (2014) Endosomes are specialized platforms for bacterial sensing and NOD2 signalling. Nature 509: 240–244. 10.1038/nature13133 24695226

[31] Ferwerda G, Girardin SE, Kullberg BJ, Le Bourhis L, de Jong DJ, et al (2005) NOD2 and toll-like receptors are nonredundant recognition systems of *Mycobacterium tuberculosis* . PLoS Pathog 1: 279–285. 16322770 10.1371/journal.ppat.0010034 PMC1291354

[32] Pandey AK, Yang Y, Jiang Z, Fortune SM, Coulombe F, et al (2009) NOD2, RIP2 and IRF5 play a critical role in the type I interferon response to *Mycobacterium tuberculosis* . PLoS Pathog 5: e1000500 10.1371/journal.ppat.1000500 19578435 PMC2698121

[33] Manzanillo PS, Shiloh MU, Portnoy DA, Cox JS (2012) *Mycobacterium tuberculosis* activates the DNA-dependent cytosolic surveillance pathway within macrophages. Cell Host Microbe 11: 469–480. 10.1016/j.chom.2012.03.007 22607800 PMC3662372

[34] Kleinnijenhuis J, Oosting M, Joosten LA, Netea MG, Van Crevel R (2011) Innate immune recognition of *Mycobacterium tuberculosis* . Clin Dev Immunol 2011:405310 10.1155/2011/405310 21603213 PMC3095423

[35] Shi S, Blumenthal A, Hickey CM, Gandotra S, Levy D, et al (2005) Expression of many immunologically important genes in *Mycobacterium tuberculosis*-infected macrophages is independent of both TLR2 and TLR4 but dependent on IFN-alphabeta receptor and STAT1. J Immunol 175: 3318–3328. 16116224 10.4049/jimmunol.175.5.3318

[36] Wong KW, Jacobs WR Jr. (2011) Critical role for NLRP3 in necrotic death triggered by *Mycobacterium tuberculosis* . Cell Microbiol 13: 1371–1384. 10.1111/j.1462-5822.2011.01625.x 21740493 PMC3257557

[37] Dorhoi A, Nouailles G, Jorg S, Hagens K, Heinemann E, et al (2012) Activation of the NLRP3 inflammasome by *Mycobacterium tuberculosis* is uncoupled from susceptibility to active tuberculosis. Eur J Immunol 42: 374–384. 10.1002/eji.201141548 22101787

[38] Watson RO, Manzanillo PS, Cox JS (2012) Extracellular M. tuberculosis DNA targets bacteria for autophagy by activating the host DNA-sensing pathway. Cell 150: 803–815. 10.1016/j.cell.2012.06.040 22901810 PMC3708656

[39] Romagnoli A, Etna MP, Giacomini E, Pardini M, Remoli ME, et al (2012) ESX-1 dependent impairment of autophagic flux by *Mycobacterium tuberculosis* in human dendritic cells. Autophagy 8: 1357–1370. 10.4161/auto.20881 22885411 PMC3442882

[40] Sokolovska A, Becker CE, Ip WK, Rathinam VA, Brudner M, et al (2013) Activation of caspase-1 by the NLRP3 inflammasome regulates the NADPH oxidase NOX2 to control phagosome function. Nat Immunol 14: 543–553. 10.1038/ni.2595 23644505 PMC3708594

[41] Cebrian I, Visentin G, Blanchard N, Jouve M, Bobard A, et al (2011) Sec22b regulates phagosomal maturation and antigen crosspresentation by dendritic cells. Cell 147: 1355–1368. 10.1016/j.cell.2011.11.021 22153078

[42] Brodin P, de Jonge MI, Majlessi L, Leclerc C, Nilges M, et al (2005) Functional analysis of early secreted antigenic target-6, the dominant T-cell antigen of *Mycobacterium tuberculosis*, reveals key residues involved in secretion, complex formation, virulence, and immunogenicity. J Biol Chem 280: 33953–33959. 16048998 10.1074/jbc.M503515200

[43] Divangahi M, Chen M, Gan H, Desjardins D, Hickman TT, et al (2009) *Mycobacterium tuberculosis* evades macrophage defenses by inhibiting plasma membrane repair. Nat Immunol 10: 899–906. 10.1038/ni.1758 19561612 PMC2730354

[44] Stanley SA, Johndrow JE, Manzanillo P, Cox JS (2007) The Type I IFN Response to infection with *Mycobacterium tuberculosis* requires ESX-1-mediated secretion and contributes to pathogenesis. J Immunol 178: 3143–3152. 17312162 10.4049/jimmunol.178.5.3143

[45] Conzelmann KK (2005) Transcriptional activation of alpha/beta interferon genes: interference by nonsegmented negative-strand RNA viruses. J Virol 79: 5241–5248. 15827138 10.1128/JVI.79.9.5241-5248.2005 PMC1082782

[46] Mayer-Barber KD, Barber DL, Shenderov K, White SD, Wilson MS, et al (2010) Caspase-1 independent IL-1beta production is critical for host resistance to *Mycobacterium tuberculosis* and does not require TLR signaling in vivo. J Immunol 184: 3326–3330. 10.4049/jimmunol.0904189 20200276 PMC3420351

[47] Hackam DJ, Rotstein OD, Zhang W, Gruenheid S, Gros P, et al (1998) Host resistance to intracellular infection: mutation of natural resistance-associated macrophage protein 1 (Nramp1) impairs phagosomal acidification. J Exp Med 188: 351–364. 9670047 10.1084/jem.188.2.351 PMC2212455

[48] Vidal MJ, Stahl PD (1993) The small GTP-binding proteins Rab4 and ARF are associated with released exosomes during reticulocyte maturation. Eur J Cell Biol 60: 261–267. 8330623

[49] Forbes JR, Gros P (2001) Divalent-metal transport by NRAMP proteins at the interface of host-pathogen interactions. Trends Microbiol 9: 397–403. 11514223 10.1016/s0966-842x(01)02098-4

[50] Lang T, Prina E, Sibthorpe D, Blackwell JM (1997) Nramp1 transfection transfers Ity/Lsh/Bcg-related pleiotropic effects on macrophage activation: influence on antigen processing and presentation. Infect Immun 65: 380–386. 9009286 10.1128/iai.65.2.380-386.1997 PMC174606

[51] Gao LY, Guo S, McLaughlin B, Morisaki H, Engel JN, et al (2004) A mycobacterial virulence gene cluster extending RD1 is required for cytolysis, bacterial spreading and ESAT-6 secretion. Mol Microbiol 53: 1677–1693. 15341647 10.1111/j.1365-2958.2004.04261.x

[52] Martin CJ, Booty MG, Rosebrock TR, Nunes-Alves C, Desjardins DM, et al (2012) Efferocytosis is an innate antibacterial mechanism. Cell Host Microbe 12: 289–300. 10.1016/j.chom.2012.06.010 22980326 PMC3517204

[53] Barel M, Charbit A (2013) *Francisella tularensis* intracellular survival: To eat or to die. Microbes Infect 25: 00206–00202. 10.1016/j.micinf.2013.09.009 24513705

[54] Cossart P (2011) Illuminating the landscape of host-pathogen interactions with the bacterium *Listeria monocytogenes* . Proc Natl Acad Sci U S A 108: 19484–19491 10.1073/pnas.1112371108 22114192 PMC3241796

[55] Armstrong JA, Hart PD (1971) Response of cultured macrophages to *Mycobacterium tuberculosis*, with observations on fusion of lysosomes with phagosomes. J Exp Med 134: 713–740. 15776571 10.1084/jem.134.3.713 PMC2139093

[56] Leake ES, Myrvik QN, Wright MJ (1984) Phagosomal membranes of *Mycobacterium bovis* BCG-immune alveolar macrophages are resistant to disruption by *Mycobacterium tuberculosis* H37Rv. Infect Immun 45: 443–446. 6430807 10.1128/iai.45.2.443-446.1984 PMC263253

[57] McDonough KA, Kress Y, Bloom BR (1993) Pathogenesis of tuberculosis: interaction of *Mycobacterium tuberculosis* with macrophages. Infect Immun 61: 2763–2773. 8514378 10.1128/iai.61.7.2763-2773.1993 PMC280919

[58] Creasey EA, Isberg RR (2014) Maintenance of vacuole integrity by bacterial pathogens. Curr Opin Microbiol 17:46–52. 10.1016/j.mib.2013.11.005 24581692 PMC4009691

[59] Myrvik QN, Leake ES, Wright MJ (1984) Disruption of phagosomal membranes of normal alveolar macrophages by the H37Rv strain of *Mycobacterium tuberculosis*. A correlate of virulence. Am Rev Respir Dis 129: 322–328. 6421212

[60] Yoshimori T, Yamamoto A, Moriyama Y, Futai M, Tashiro Y (1991) Bafilomycin A1, a specific inhibitor of vacuolar-type H(+)-ATPase, inhibits acidification and protein degradation in lysosomes of cultured cells. J Biol Chem 266: 17707–17712. 1832676

[61] Mwandumba HC, Russell DG, Nyirenda MH, Anderson J, White SA, et al (2004) *Mycobacterium tuberculosis* resides in nonacidified vacuoles in endocytically competent alveolar macrophages from patients with tuberculosis and HIV infection. J Immunol 172: 4592–4598. 15034077 10.4049/jimmunol.172.7.4592

[62] Russell DG, Mwandumba HC, Rhoades EE (2002) *Mycobacterium* and the coat of many lipids. J Cell Biol 158: 421–426. 12147678 10.1083/jcb.200205034 PMC2173834

[63] Kondo E, Yasuda T, Kanai K (1982) Electron microscopic demonstration of close contact between intracellular mycobacteria and the phagosomal membrane. Jpn J Med Sci Biol 35: 197–201. 6818395 10.7883/yoken1952.35.197

[64] Merckx JJ, Brown AL Jr., Karlson AG (1964) An electron-microscopic study of experimental infections with Acid-Fast Bacilli. Am Rev Respir Dis 89: 485–496. 14139316 10.1164/arrd.1964.89.4.485

[65] Moreira AL, Wang J, Tsenova-Berkova L, Hellmann W, Freedman VH, et al (1997) Sequestration of *Mycobacterium tuberculosis* in tight vacuoles in vivo in lung macrophages of mice infected by the respiratory route. Infect Immun 65: 305–308. 8975928 10.1128/iai.65.1.305-308.1997 PMC174592

[66] Pym AS, Brodin P, Majlessi L, Brosch R, Demangel C, et al (2003) Recombinant BCG exporting ESAT-6 confers enhanced protection against tuberculosis. Nat Med 9: 533–539. 12692540 10.1038/nm859

[67] Brodin P, Majlessi L, Brosch R, Smith D, Bancroft G, et al (2004) Enhanced protection against tuberculosis by vaccination with recombinant *Mycobacterium microti* vaccine that induces T cell immunity against region of difference 1 antigens. J Infect Dis 190: 115–122. 15195250 10.1086/421468

[68] Rybniker J, Chen JM, Sala C, Hartkoorn RC, Vocat A, et al (2014) Anticytolytic screen identifies inhibitors of mycobacterial virulence protein secretion. Cell Host Microbe 16: 538–548. 10.1016/j.chom.2014.09.008 25299337

[69] Christophe T, Jackson M, Jeon HK, Fenistein D, Contreras-Dominguez M, et al (2009) High content screening identifies decaprenyl-phosphoribose 2' epimerase as a target for intracellular antimycobacterial inhibitors. PLoS Pathog 5: e1000645 10.1371/journal.ppat.1000645 19876393 PMC2763345

[70] Astarie-Dequeker C, Le Guyader L, Malaga W, Seaphanh FK, Chalut C, et al (2009) Phthiocerol dimycocerosates of *M*. *tuberculosis* participate in macrophage invasion by inducing changes in the organization of plasma membrane lipids. PLoS Pathog 5: e1000289 10.1371/journal.ppat.1000289 19197369 PMC2632888

[71] Brodin P, Poquet Y, Levillain F, Peguillet I, Larrouy-Maumus G, et al (2010) High content phenotypic cell-based visual screen identifies *Mycobacterium tuberculosis* acyltrehalose-containing glycolipids involved in phagosome remodeling. PLoS Pathog 6: e1001100 10.1371/journal.ppat.1001100 20844580 PMC2936551

[72] MacGurn JA, Cox JS (2007) A genetic screen for *Mycobacterium tuberculosis* mutants defective for phagosome maturation arrest identifies components of the ESX-1 secretion system. Infect Immun 75: 2668–2678. 17353284 10.1128/IAI.01872-06 PMC1932882

[73] Stewart GR, Patel J, Robertson BD, Rae A, Young DB (2005) Mycobacterial mutants with defective control of phagosomal acidification. PLoS Pathog 1: 269–278. 16322769 10.1371/journal.ppat.0010033 PMC1291353

[74] Vandal OH, Pierini LM, Schnappinger D, Nathan CF, Ehrt S (2008) A membrane protein preserves intrabacterial pH in intraphagosomal *Mycobacterium tuberculosis* . Nat Med 14: 849–854. 10.1038/nm.1795 18641659 PMC2538620

[75] Sayes F, Sun L, Di Luca M, Simeone R, Degaiffier N, et al (2012) Strong immunogenicity and cross-reactivity of *Mycobacterium tuberculosis* ESX-5 type VII secretion-encoded PE-PPE proteins predicts vaccine potential. Cell Host Microbe 11: 352–363. 10.1016/j.chom.2012.03.003 22520463

[76] Livak KJ, Schmittgen TD (2001) Analysis of relative gene expression data using real-time quantitative PCR and the 2(-Delta Delta C(T)) Method. Methods 25: 402–408. 11846609 10.1006/meth.2001.1262

