# Supplementary figures and images for: Cytosolic Access of Mycobacterium tuberculosis: Critical Impact of Phagosomal Acidification Control and Demonstration of Occurrence In Vivo
Source: PLoS Pathog. 2015 Feb 6;11(2):e1004650. doi: 10.1371/journal.ppat.1004650 (PMC4450080; doi:10.1371/journal.ppat.1004650)

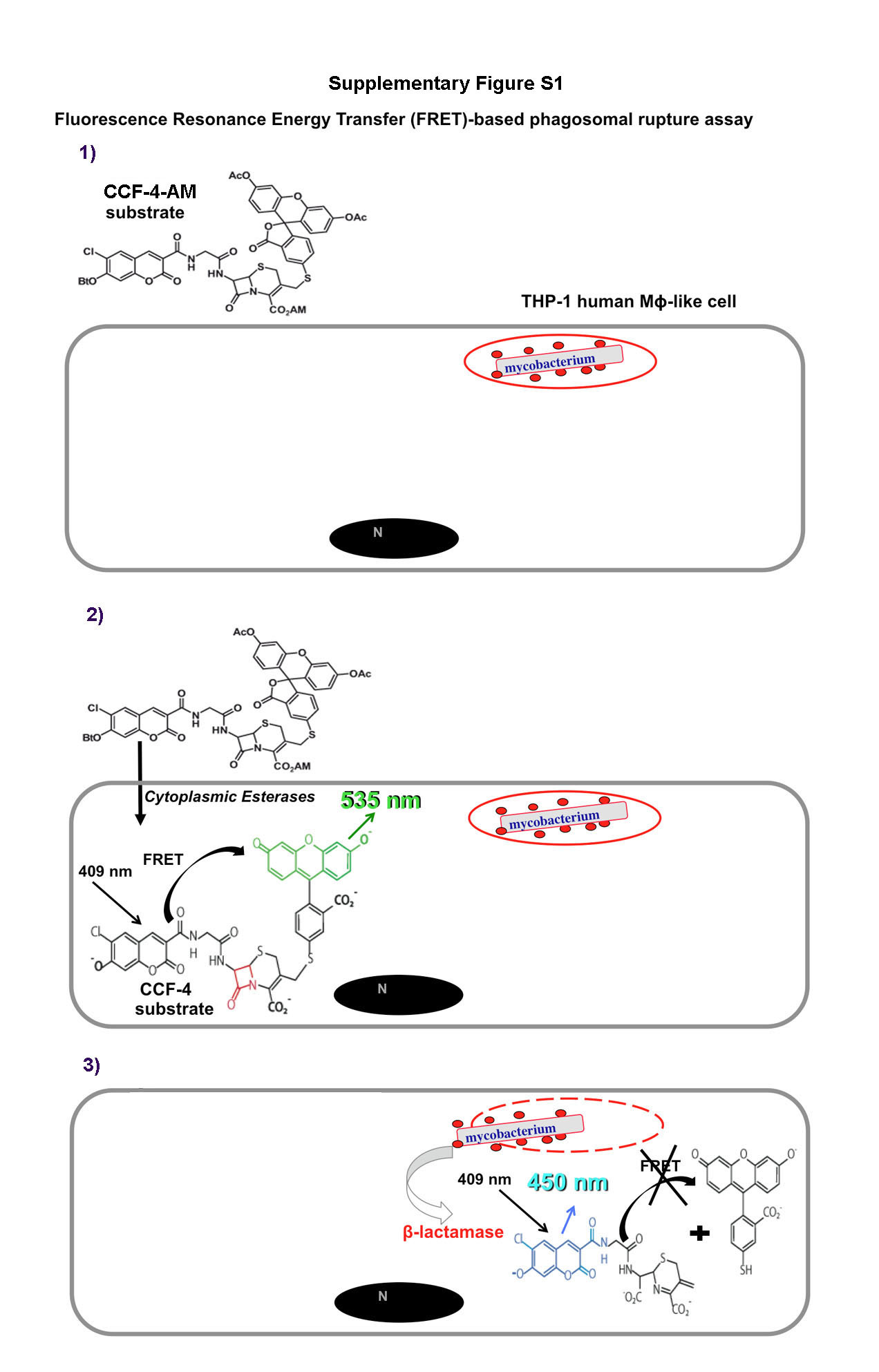

Supplement: S1 Fig — In step 1, CCF4-AM (Life Technologies) represents a lipophilic, esterified form of the CCF4 substrate, which allows it to readily enter cells. As shown for step 2, upon entry, cleavage by endogenous cytoplasmic esterases rapidly converts CCF4-AM into its negatively charged form, CCF4, which is retained in the cytosol and thus cannot enter into the different cell organelles, including phagosomes containing bacteria. In case the bacteria remain engulfed in the intact phagosome, the endogenous bacterial beta-lactamase can not reach the CCF-4 substrate and upon stimulation at ~ 409 nm, CCF-4 shows Fluorescence Resonance Energy Transfer (FRET) that leads to emmision of “green” light at ~ 535 nm. As shown for step 3, in case of phagosomal rupture and cytosolic contact the mycobacterial β-lactamase (shown as red dots linked to the bacterium) gets in contact with the CCF-4 substrate trapped in the cytosol, and is inducing cleavage of the substrate and inhibiting FRET, thereby leading to emission of “blue” light at ~ 450 nm. (JPG) [file ppat.1004650.s001.jpg]

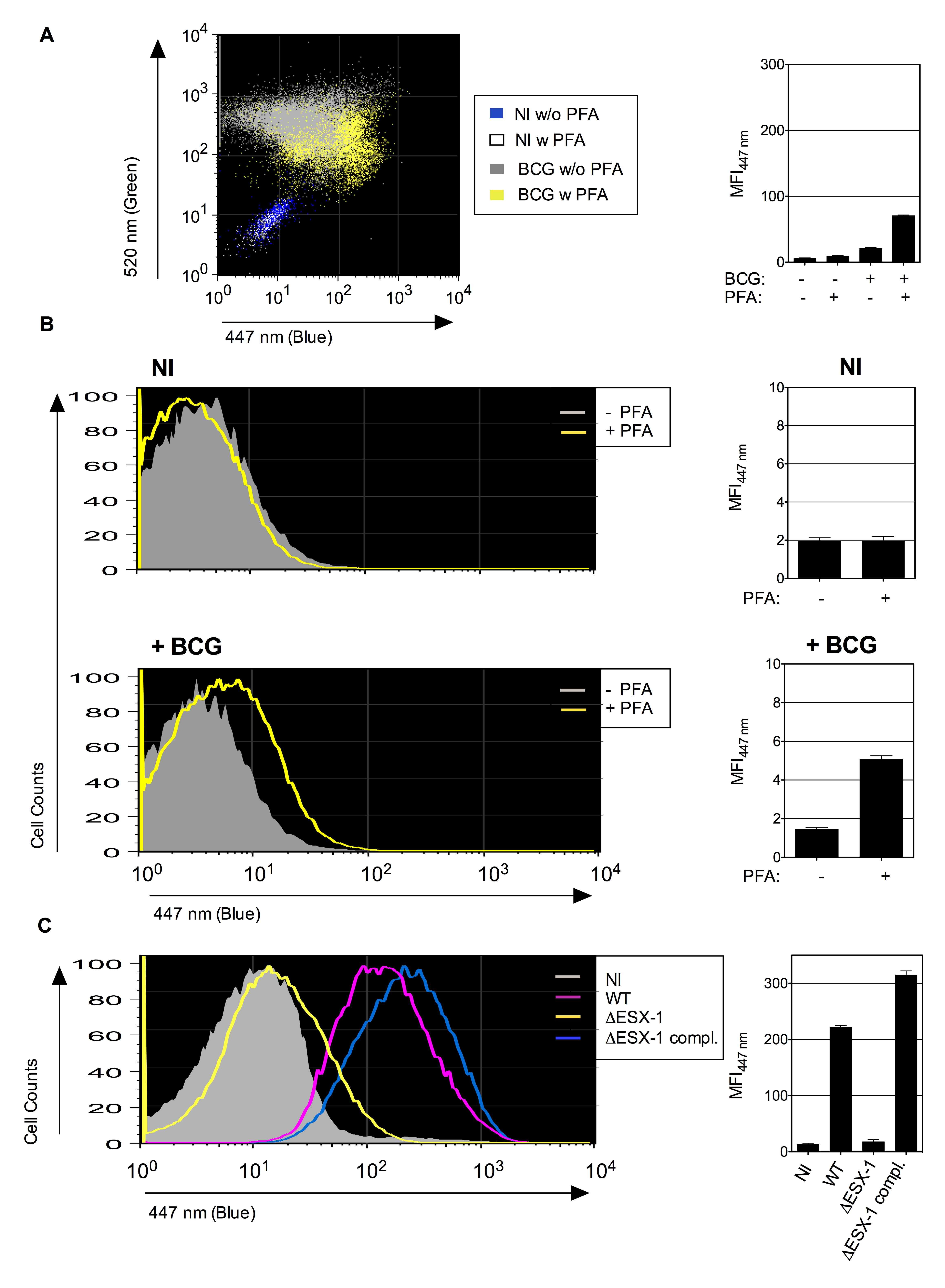

Supplement: S2 Fig — PFA fixation of mycobacteria-infected cells results in some levels of CCF-4 bleu shift. (A) PFA fixation of 1 cells or (B) BM-DC infected with M. bovis BCG, deficient in ESX-1, results in low CCF-4 shift to blue, which is plausibly linked to a small leak of β-lactamase activity into the cytosol soon after the cell fixation prior to signal acquisition. However, these levels of shift are ten to hundred of times lower compared to those observed with cells infected with ESX-1-sufficient mycobacteria. (C) Complementation of ΔESX-1 H37Rv mutant with complete ESX-1 genomic region restores the capacity of Mtb to induce phagosomal rupture. Phagosomal rupture induced by WT, ΔESX-1 or ΔESX-1 complemented with complete ESX-1-region in infected BM-DC (MOI = 1), as determined by the profile of green vs. blue CCF-4 signals at 5 dpi. MFI447 nm values in different infected BM-DC groups are indicated. (JPG) [file ppat.1004650.s002.jpg]

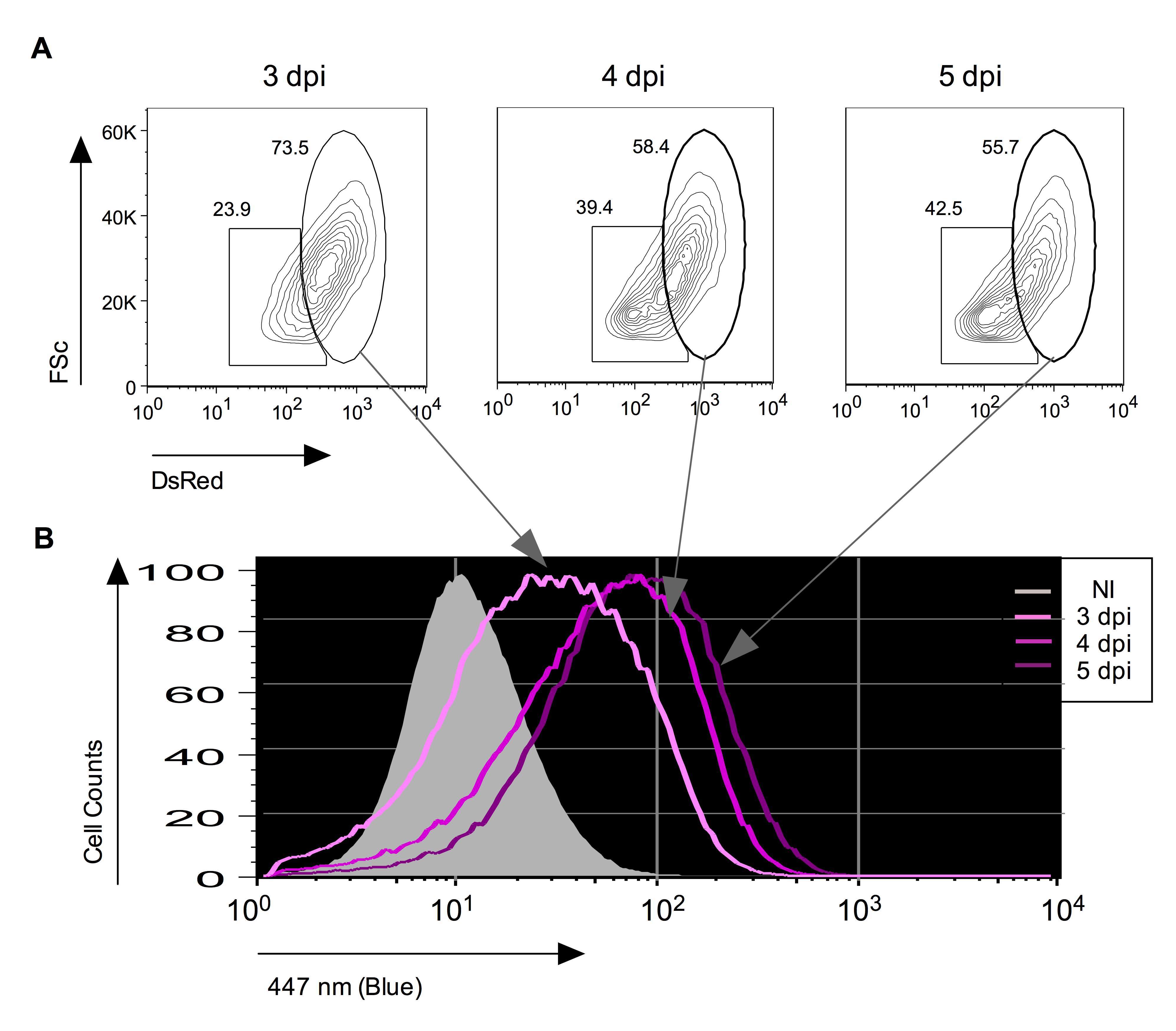

Supplement: S3 Fig — Cultures of BM-DC were infected with DsRed-WT Mtb (MOI = 1) and the cells were analyzed from 3 to 5 dpi. (A) Cells containing DsRed Mtb were gated and (B) their CCF-4 blue signal were overlayed and compared to that of uninfected cells. (JPG) [file ppat.1004650.s003.jpg]

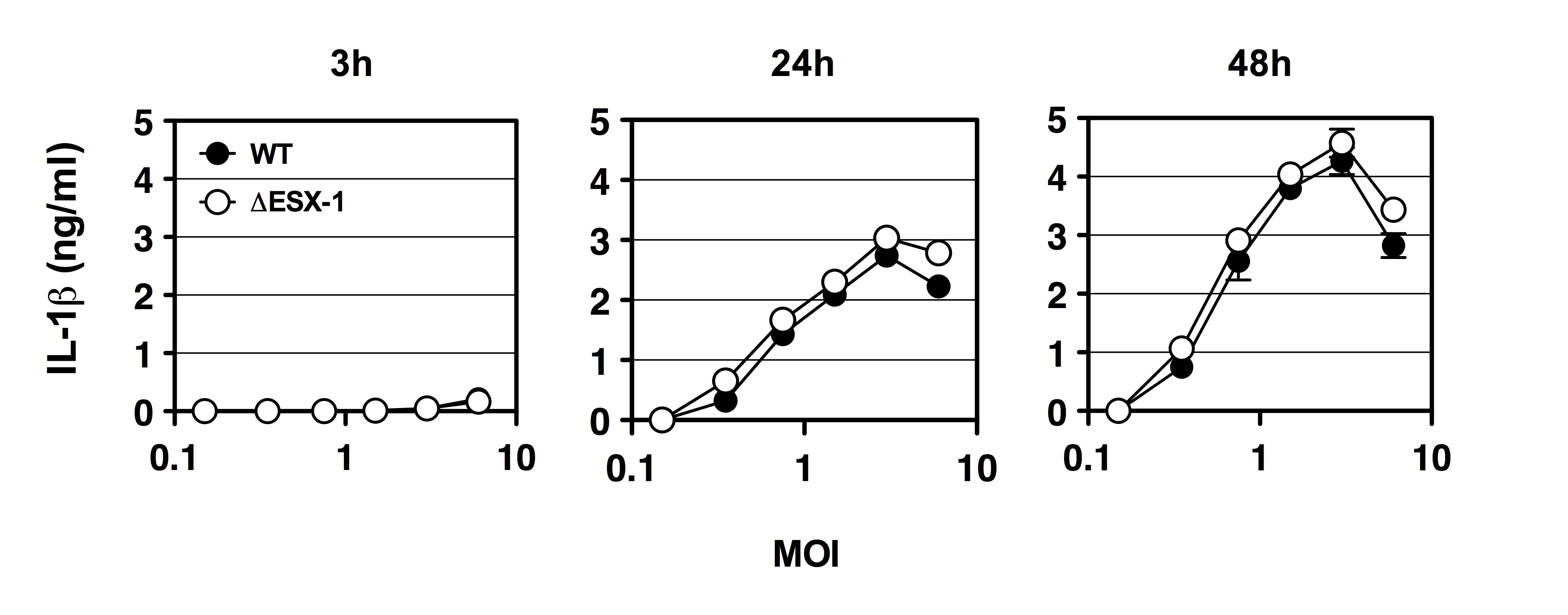

Supplement: S4 Fig — IL-1β concentrations, as quantified in the supernatants of BM-DC shown in Fig. 3, at 3, 24 and 48 h following infection with Mtb WT or ΔESX-1 at different MOI. (JPG) [file ppat.1004650.s004.jpg]

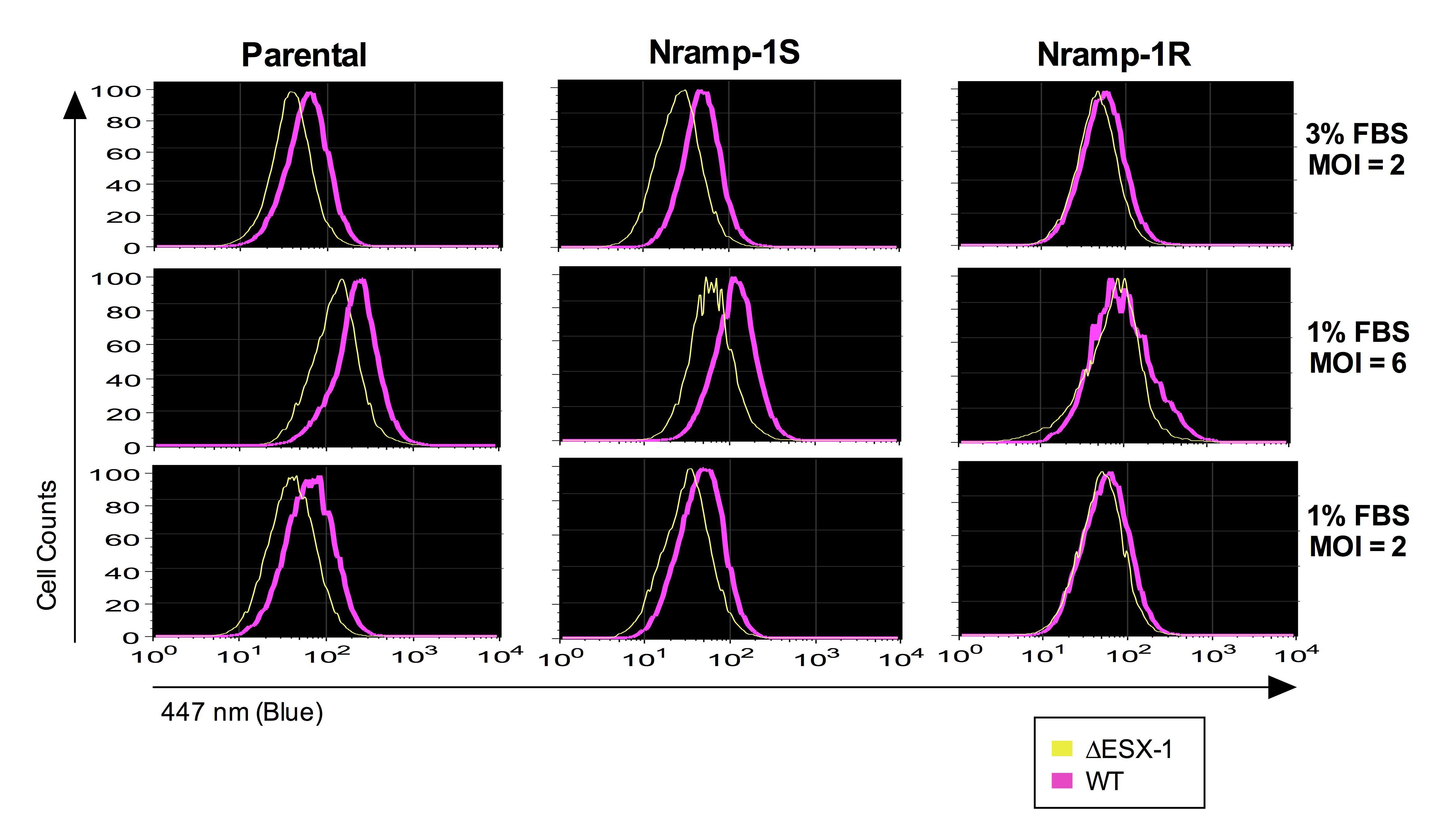

Supplement: S5 Fig — Effect of different FBS percentages in the culture medium, directly governing the rate of Raw246.7 cell proliferation and different MOI of WT or ΔESX-1 Mtb, as evaluated in Raw246.7 cells, parental or transfected with nramp-1S or nramp-1R. Shown are comparative blue CCF-4 signals. It is noteworthy that, compared to THP-1 cells, BM-DC or BM-MΦ, relatively low levels of phagosomal rupture were generally observable in Raw264.7 MΦ. Indeed, a CCF-4 blue shift is weakly detectable at 2 dpi, peaks at 3 dpi and then decreases as soon as 4 dpi. This feature seems to be linked to intense proliferative capacity of these cells despite the infection and also to their possible intense efferocytic capacity. (JPG) [file ppat.1004650.s005.jpg]

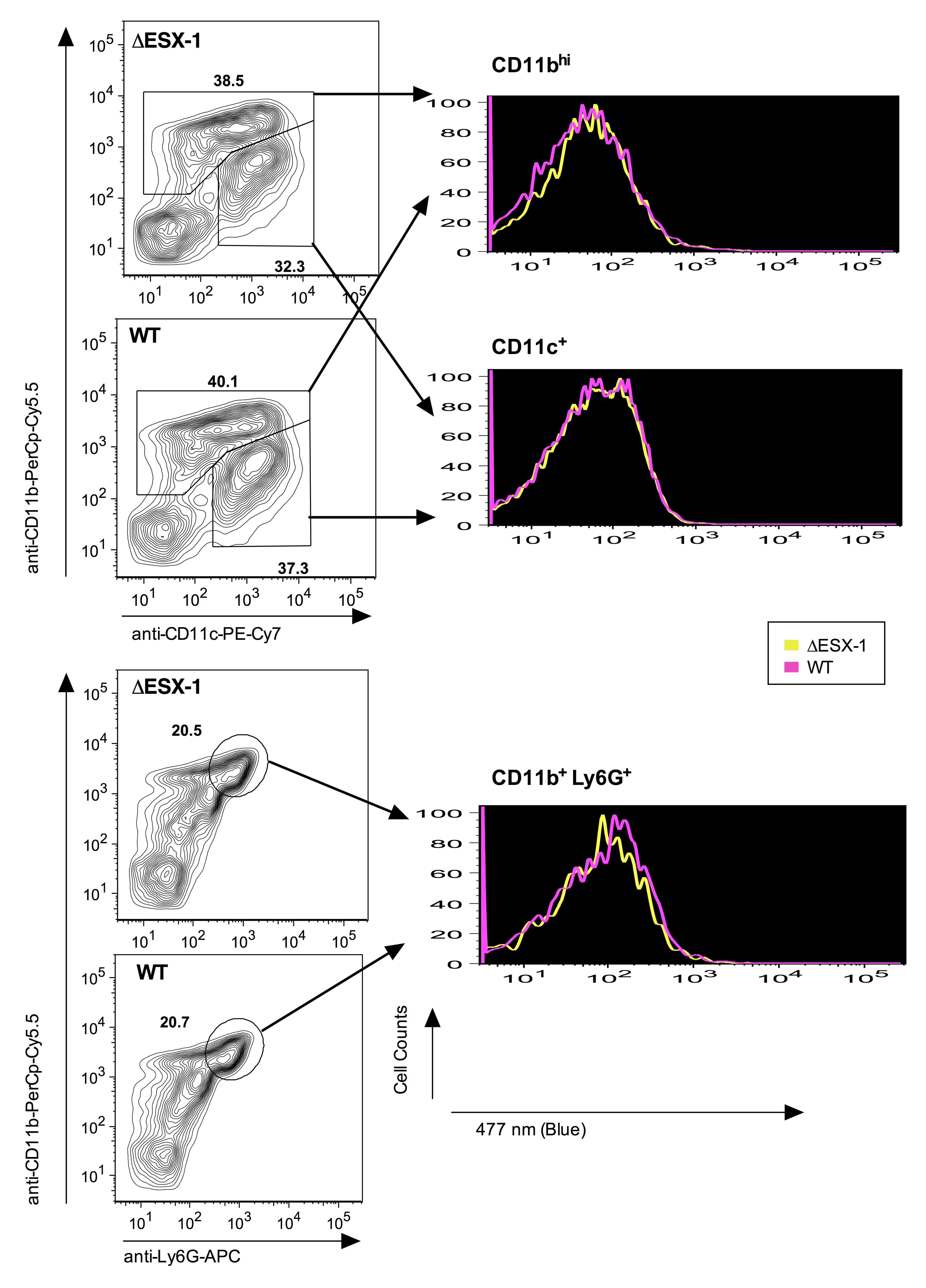

Supplement: S6 Fig — T-/B-cell deficient rag2 °/° mice were infected i.v. with 1 x 10^6^ CFU/mouse. At 1, 2 or 3 wks p.i., low density cells from the spleen were stained with CCF-4 and subsequently with cocktails of mAbs to distinguish different innate cell subsets, i.e., DC (CD11c+ CD11b+), MΦ/monocytes (CD11c—CD11b+) or neutrophils (CD11b+ Ly6G+). Shown are results obtained at 2 wks p.i.. Comparable results were obtained at 1 or 3 wks p.i. with both spleen and lung low density cells. (JPG) [file ppat.1004650.s006.jpg]

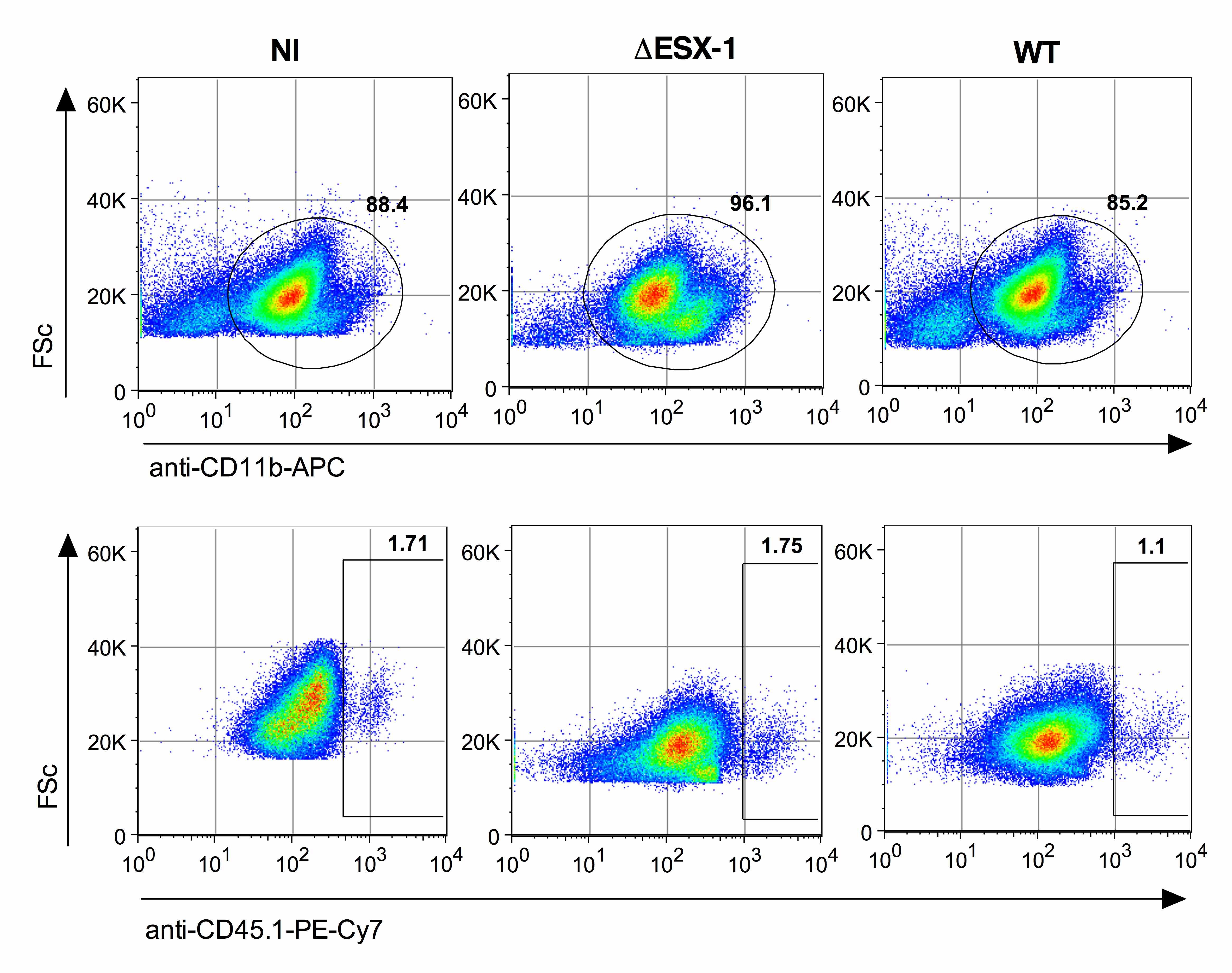

Supplement: S7 Fig — BM-DC from CD45.1 donor mice, non-infected or infected with Mtb ΔESX-1 or WT, were transferred i.n. into the CD45.2 recipients. Shown are the low-density cells recovered from the lung parenchyma of the recipients of each group at day 4 post transfer. Cells were first gated on FSc/CD11b and then for CD45.1+ cells. The comparative CCF-4 blue signal of such cells from different experimental groups are shown in Fig. 7C. (JPG) [file ppat.1004650.s007.jpg]
